# Conventional GnRH antagonist protocols versus long GnRH agonist protocol in IVF/ICSI cycles of polycystic ovary syndrome women: a systematic review and meta-analysis

**DOI:** 10.1038/s41598-022-08400-z

**Published:** 2022-03-15

**Authors:** Sally Kadoura, Marwan Alhalabi, Abdul Hakim Nattouf

**Affiliations:** 1 Department of Pharmaceutics and Pharmaceutical Technology, Faculty of Pharmacy, Damascus University, Damascus, Syrian Arab Republic; 2 Department of Embryology and Reproductive Medicine, Faculty of Medicine, Damascus University, Damascus, Syrian Arab Republic; 3 Assisted Reproduction Unit, Orient Hospital, Damascus, Syrian Arab Republic

**Keywords:** Infertility, Health care

## Abstract

Gonadotropin-releasing hormone (GnRH) analogues are commonly used in clinical practice to prevent premature luteinizing hormone (LH) surge during In-Vitro Fertilization/ Intra-Cytoplasmic Sperm Injection (IVF/ICSI) cycles. This review aimed to summarize the available evidence comparing the effects of conventional GnRH antagonist protocols, the most commonly used GnRH antagonist protocols, and GnRH agonist protocols on IVF/ICSI outcomes in women with polycystic ovary syndrome (PCOS). A comprehensive electronic search was carried out in Pubmed, Cochrane CENTRAL, Scopus, Web of Science, CINAHL, TRIP, ClinicalTrials.gov and ISRCTN registry from inception until 24 November 2020 without any language or date restrictions. In addition, reference lists of eligible studies and previous meta-analyses were hand-searched to identify relevant studies. Eligible randomized controlled trials were those designed to compare the effects of conventional GnRH antagonist protocols and GnRH agonist protocols on IVF/ICSI outcomes in PCOS subjects. The Cochrane ROB 2.0 tool was used to assess the risk of bias of each study, and the GRADE assessment was used to evaluate the overall quality of evidence. Data synthesis and analyses were done using Review Manager 5.3 with the assistance of Revman Web. A random-effects model was used for all meta-analysis. Dichotomous outcomes were reported as Relative Risk (RR) and continuous outcomes as Weighted Mean Difference (WMD), both with 95% CIs. The primary outcomes were Live birth rate, Ongoing pregnancy rate, and Ovarian hyperstimulation syndrome (OHSS) rate. Other IVF outcomes were considered secondary outcomes. We included ten studies with 1214 randomized PCOS women. Using GnRH antagonist protocols led to a significantly lower OHSS rate (RR = 0.58; 95% CI: [0.44 to 0.77], *P* = 0.0002), shorter stimulation duration (WMD = − 0.91; 95% CI: [-1.45 to − 0.37] day, *P* = 0.0009), lower gonadotropin consumption (WMD = − 221.36; 95% CI: [− 332.28 to − 110.45] IU, *P* < 0.0001), lower E2 levels on hCG day (WMD = − 259.21; 95% CI: [− 485.81 to − 32.60] pg/ml, *P* = 0.02), thinner endometrial thickness on hCG day (WMD = − 0.73; 95% CI: [− 1.17 to − 0.29] mm, *P* = 0.001), and lower number of retrieved oocytes (WMD = − 1.82; 95% CI: [− 3.48 to − 0.15] oocytes, *P* = 0.03). However, no significant differences in live birth rate, ongoing pregnancy rate, clinical pregnancy rate, multiple pregnancy rate, miscarriage rate and cycle cancellation rate were seen between the GnRH antagonist protocols and the long GnRH agonist one. Although more cycles were cancelled due to poor ovarian response in the GnRH antagonist protocol (RR = 4.63; 95% CI: [1.49 to 14.41], *P* = 0.008), similar rates of cancellation due to risk of OHSS were noticed in both groups. The differences in IVF/ICSI outcomes may arise from the different patterns of gonadotropins suppression that the GnRH analogues exhibit during the early follicular phase of IVF/ICSI cycles and the divergent direct impacts of these analogues on ovaries and endometrial receptivity. The main evidence limitation was Imprecision. Conventional GnRH antagonist protocols represent a safer and more cost-effective treatment choice for PCOS women undergoing IVF/ICSI cycles than the standard long GnRH agonist protocol without compromising the IVF/ICSI clinical outcomes. The study had no sources of financial support and was prospectively registered at PROSPERO (International Prospective Register of Systematic Reviews) under registration number (CRD42021242476).

## Introduction

Polycystic ovary syndrome (PCOS) is the most common endocrine disorder among females of reproductive age^1^ and is considered the principal cause of anovulation cases referring to infertility clinics^2^. Based on the current recommendations, In-vitro Fertilization/ Intra-Cytoplasmic Sperm Injection (IVF/ICSI) can be offered to PCOS women as a third-line treatment choice after the failure of other approaches of ovulation induction^3^. However, it poses challenges considering the hormonal imbalance, obesity, hyperandrogenism, and insulin resistance observed in this population^4^. In addition, pregnancy enhances the low-grade inflammation usually noted in PCOS women, which in combination with the subclinical impairment of vascular structure and function could result in a hypoxic state that alters remodeling of spiral arteries and subsequently leads to a reduction in uterine artery impedance, a reduction in the depth of endovascular trophoblast and eventually abnormal placentation^5,6^. All of this puts PCOS women who conceived using IVF cycles at higher risk of developing adverse pregnancy and neonatal complications like miscarriage, gestational diabetes mellitus, pregnancy-induced hypertension, preterm birth, and giving birth of large-for-gestational-age babies^4,7^. Since the 1980s, Gonadotropin-releasing hormone (GnRH) agonists have been added to the controlled ovarian hyperstimulation (COS) protocols of IVF/ICSI to prevent premature luteinizing hormone (LH) surge while the follicles are still immature, resulting in a higher clinical pregnancy rate (CPR), lower cycle cancellation rate (CCR) and higher number of the retrieved oocytes^8,9^. However, due to the flare-up phase, GnRH agonist protocols are associated with some disadvantages, e.g., prolonged protocol duration, higher risk of formation ovarian cysts, and developing hypo-estrogenic side effects^10,11^. In addition, their use is associated with an increased risk of developing ovarian hyperstimulation syndrome (OHSS)^8^, an iatrogenic complication of COS characterized by cystic enlargement of the ovaries and fluid shifting from the intravascular to the third space due to increased capillary permeability and ovarian neo-angiogenesis^12^. This harmful effect may be more detrimental among PCOS women, who are already prone to developing OHSS^4,7,13^ due to the increased antral follicular counts, increased anti-müllerian hormone (AMH) levels, and increased estradiol levels, which exaggerates their response and sensibility to COS^14,15^. Thus, attempting to introduce more patient-friendly and cost-effective protocols, GnRH antagonists have been introduced to routine practice as alternatives to GnRH agonists. Unlike the indirect pituitary suppression induced by GnRH agonists, GnRH antagonists immediately and competitively occupy the GnRH receptors^16^, which helps to overcome the unfavorable effects of GnRH agonist protocols^11^. GnRH antagonists are usually scheduled during COS based on the progression of follicles development; detecting a leading follicle ≥ 12–14 mm diameter (Flexible protocol)^17^, or they are used from Day 5/ Day 6 of stimulation onward (Fixed protocol)^18,19^. However, recent research revealed that high gonadotropins levels during the early follicular phase of COS might have harmful effects on IVF/ICSI outcomes, as unsuppressed follicle-stimulation hormone (FSH) leads to an uncoordinated development of FSH‐sensitive follicles and a reduction in viable oocytes number^20,21^. While unsuppressed LH enhances estradiol (E2) production, in consequence, higher exposure of the genital tract to LH, E2, and progesterone might adversely affect the implantation rate mainly by altering endometrial receptivity^22^. Thus, new GnRH antagonist protocols were introduced to suppress gonadotropin levels, not only during the mid-follicular phase but also during the early follicular phase either as an Early-late Flexible protocol^23,24^ or an Early Fixed protocol^25–27^. However, Conventional GnRH antagonist protocols are still more commonly used in clinics than the Early GnRH antagonist ones. Several meta-analyses^28–32^ compared the safety and efficacy of treating PCOS women with GnRH antagonist protocols and the standard Long GnRH agonist protocol. However, all of them have included all available GnRH antagonist protocols; Early GnRH antagonist protocols, and Conventional GnRH antagonist ones. Moreover, most previous reviews^28–30,32^ have included different triggering agents in the GnRH antagonist group; GnRH agonist trigger and human chorionic gonadotropin (hCG) trigger. A recent Cochrane review^33^ demonstrated that using GnRH agonists for final oocyte maturation triggering is associated with a lower live birth rate; lower ongoing pregnancy rate; lower incidence of mild, moderate, and severe OHSS; and a higher rate of early miscarriage compared with hCG trigger in fresh cycles. Thus, we conducted this systematic review and meta-analysis to summarize the available evidence comparing the effects of Conventional GnRH antagonist protocols, the most commonly used GnRH antagonist protocols, and GnRH agonist protocols on IVF/ICSI outcomes in women with PCOS.

## Materials and methods

### Protocol and registration

This review was performed in accordance with relevant guidelines and regulations. The preferred reporting items for systematic reviews and meta-analysis (PRISMA) guidelines 2020^34^were followed to assure transparent reporting, see Supplementary Tables S1 and S2. The protocol of this study was registered at PROSPERO under registration number (CRD42021242476).

### Eligibility criteria

The Participants, Intervention, Comparison, Outcomes, and Studies (PICOS) framework was as follow:

- Participants: PCOS women undergoing controlled ovarian hyperstimulation as part of an IVF or ICSI programme.
- Intervention: Conventional GnRH antagonist protocols.
- Comparison: GnRH agonist protocols.
- Outcomes: Live birth rate (LBR) per randomized woman, Ongoing pregnancy rate (OPR) per randomized woman, Ovarian hyperstimulation syndrome (OHSS) rate per woman randomized, Clinical pregnancy rate (CPR) per randomized woman, Multiple pregnancy rate (MPR) per randomized woman, Miscarriage rate (MR) per randomized woman, Cycle cancellation rate (CCR) per randomized woman, Stimulation duration (days), Gonadotropin consumption (IUs), E2 levels on hCG day, Endometrial thickness (in millimeters), Number of retrieved oocytes.
- Study design: RCT.
- Limits: human studies.

Studies were only included when data of one outcome of the outcomes of interest at least were available in a manner that allowed their inclusion in a meta-analysis.

### Exclusion criteria

- Meta-analysis, systematic literature reviews, narrative reviews, case reports, observational studies, animal studies, in-vitro studies.
- RCTs comparing Early GnRH antagonist protocols with GnRH agonist protocols.
- Studies reporting the desired outcomes per cycle and not per woman. (study authors were contacted to request the data per woman. If no response was obtained, the study was excluded).
- Studies comparing more than one variable, like those comparing GnRH agonist versus hCG for triggering, in addition to comparing GnRH antagonist with GnRH agonist for suppression.

Regarding conference abstracts, if there was not enough information to decide their eligibility for inclusion or their results were not available numerically, study authors were contacted to provide missing information and data. If no response was obtained, the study was excluded.

### Information sources and search strategies

A comprehensive electronic search was carried out in Pubmed, Cochrane Central Register of Controlled Trials (CENTRAL), Scopus, Web of Science, Cumulative Index to Nursing and Allied Health Literature (CINAHL), Turning Research Into Practice (TRIP), ClinicalTrials.gov and ISRCTN registry from inception up until 24 November 2020 without any language or date restrictions. In addition, reference lists of eligible studies and previous meta-analyses were hand-searched to identify relevant studies. The search terms were modified when required for each database. The full-search strategy is reported in Supplementary Table S3.

### Selection process

Duplication removal was conducted using two software independently, Mendeley Desktop 1.19.4 software (Glyph & Cog, LLC) and Microsoft Office Excel 2016 (Microsoft Corporation), to limit false duplicate, i.e. incorrectly identified as duplicate. Two authors (S.K. and A.N.) independently double screened articles by title and abstract. Subsequently, the same reviewers independently completed full-text screening of all potentially eligible studies and examined their compliance with the inclusion criteria. Any discrepancies during the study selection process were resolved by consultation with the third reviewer (M.A.). Where required, study investigators were contacted to clarify study eligibility.

### Data collection process and data items

A data extraction form was created in Microsoft Office Excel 2016 (Microsoft Corporation) to facilitate the retrieval and storage of relevant data. The following information was sought from included studies: Publication details (first author's name, year of publication), Methods (study design, study duration, country of conduct), Participant’s characteristics (inclusion criteria, exclusion criteria, baseline characteristics, number of randomized women, number of excluded women after randomization, number of women lost to follow-up/ drop-out with reasons). Intervention/Comparison (Protocol used, type of GnRH antagonist/agonist used and when it was started, protocol pre-treatment drugs, stimulator used (type, when it was started, starting dose), trigger used, luteal phase support, transfer day), and all data that would allow the estimation of the effects of interest were extracted. In addition, we extracted information about the randomization process, allocation concealment, blinding, type of analysis, funding, OHSS diagnosis criteria and cycle cancellation criteria. Two authors (S.K. and A.N) independently extracted the data, and any disagreements were resolved by discussion with the third author (M.A). Where a study had multiple publications, the authors collated multiple reports of the same study so that each study rather than each report was the unit of interest in the review, and such study had a single study ID with multiple references^35^.

Outcomes of interest:

- Primary outcomes:
  - Live birth rate (LBR) per woman randomized, defined as delivery of a live fetus after 20 completed weeks of gestation.
  - Ongoing pregnancy rate (OPR) per randomized woman, defined as the number of pregnancies beyond 12 weeks' gestation per number of randomized women.
  - Ovarian hyperstimulation syndrome (OHSS) rate per randomized woman.
- Secondary outcomes:
  - Clinical pregnancy rate (CPR) per randomized woman, defined as the number of pregnancies with at least one gestational sac ± fetal heartbeat at transvaginal ultrasound per number of randomized women.
  - Multiple pregnancy rate (MPR) per randomized woman, defined as the number of pregnancies with two or more gestational sacs on transvaginal ultrasound per number of randomized women.
  - Miscarriage rate (MR) per randomized woman, defined as the number of pregnancies ending in the spontaneous loss of the embryo or fetus before 20 weeks' gestation per number of randomized women.
  - Cycle cancellation rate (CCR) per woman randomized.
  - Stimulation duration (days).
  - Gonadotropin consumption (IUs).
  - E2 levels on hCG day.
  - Endometrial thickness (in millimeters) by ultrasound scan on hCG day.
  - Number of retrieved oocytes.

### Assessment of risk of bias in included studies

Two authors (S.K. and M.A.) independently assessed the risk of bias of each study using the revised Cochrane risk of bias tool for randomized controlled trials (ROB 2.0)^36,37^. Using ROB 2.0 tool, each study is judged to have either low risk, some concerns, or a high risk of bias for the studied outcome. Any disagreement was resolved by consultation with a third reviewer (N.A.). The risk of bias tool addresses the following domains: Bias arising from the randomization process; Bias due to deviations from intended interventions; Bias due to missing outcome data; Bias in the measurement of the outcome; Bias due to selection of the reported result and overall bias. For each domain, a series of signalling questions with the answers (yes, probably yes, no information, probably no, no) determine the risk of bias (low risk, some concerns and high risk). The overall risk of bias generally corresponds to the worst risk of bias in any of the domains. However, if a study is judged to have “some concerns” risk of bias for multiple domains, it might be judged as at high risk of bias overall^36,37^.

### Summary measures

Dichotomous outcomes were reported as Relative Risk (Risk ratio, RR) and continuous outcomes as Weighted Mean Difference (WMD), both with 95% CIs. When overall results were significant in dichotomous outcomes, the number needed to treat for an additional beneficial outcome (NNTB) and the number needed to treat for an additional harmful outcome (NNTH) were computed from RR based on the Cochrane recommendations by combining the risk ratio with an estimate of the prevalence of the event in the control group of the trials^7^.

### Dealing with missing data

We analyzed data on an intention‐to‐treat basis as far as possible. If any information was missing, the corresponding authors were notified twice via an e-mail to retrieve the missing data. If the authors did not respond within two weeks of the first contact, we assumed that the outcome did not occur in dichotomous outcomes (e.g. clinical pregnancy rate), while we analyzed only available data in continuous ones. If the data were reported as median with (Q25, Q75) or (minimum value, maximum value) and were suspected to be skewed, we contacted study authors to request the results in mean and standard deviation (SD). If no response was obtained, the Box-Cox method of McGrath et al.^38^ to estimate the mean and SD was used. Where data presented graphically, we contacted the study authors to request the results in mean and SD. If no response was obtained, WebPlotDigitizer (Ankit Rohatgi. WebPlotDigitizer. Version 4.4. 2020. Available at https://automeris.io/WebPlotDigitizer) was used to extract this information. When the miscarriage rate measured at a “12 weeks’ gestation” time-point, and both the number of clinical pregnancies and the number of miscarriage were available, we calculated the ongoing pregnancy rate as [number of clinical pregnancies—number of miscarriage/ number of randomized woman]. Similarly, when the number of clinical pregnancies and the number of ongoing pregnancies were available, we calculated the miscarriage rate as [number of clinical pregnancies—number of ongoing pregnancies/number of randomized woman]. It is worth noting that we considered cycle cancellation cases as missing outcome data for the following outcomes: live birth rate, clinical pregnancy rate, ongoing pregnancy rate, multiple pregnancy rate and miscarriage rate since the participants no longer capable of experiencing those events.

### Data synthesis

Data synthesis and analyses were done using the Review Manager 5.3; Review Manager (RevMan). Version 5.3. Copenhagen: The Nordic Cochrane Centre, The Cochrane Collaboration, 2014, with the assistance of Revman Web; Review Manager Web (RevMan Web). Version (2.4.1). The Cochrane Collaboration, (2021). Available at revman.cochrane.org; to create forest plots with ROB assessment since Review Manager v.5.3 only supports ROB 1 tool. A random-effects model was used for all meta-analysis. Heterogeneity amongst the included studies was evaluated qualitatively; through visual inspection of forest plots and comparing the characteristics of included studies, and quantitatively; using the χ^2^ test of heterogeneity and the I^2^ statistic. Subgroup analyses were performed to detect potential sources of heterogeneity based on the type of antagonist protocol used (Flexible VS Fixed). The following sensitivity analyses were also performed to determine whether the conclusions are robust to arbitrary decisions made regarding the eligibility criteria and analysis process: the removal of high risk of bias studies and estimated outcome data (converting medians and (Q25, Q75) or medians and (minimum value, maximum value) to means ± standard deviations, and estimating the outcome data from a graph).

### Publication bias assessment

Given the difficulty of detecting and correcting publication bias and other reporting biases, we aimed to minimize their potential impact by ensuring a comprehensive search for eligible studies and being alert to duplication of data. If ≥ ten studies were included in a comparison, a funnel plot was produced to graphically explore the possibility of small-study effects (a tendency for estimates of the intervention effect to be more beneficial in smaller studies) as when there are fewer studies, the power of the tests is weak^39^.

### Assessment of certainty of evidence

The Grading of Recommendations Assessment, Development, and Evaluation (GRADE) assessment^40^ was used to assess the overall quality of evidence for each outcome by two authors (S.K. and M.A.) independently, and a “Summary of findings” table was created using GradePro GDT [GRADEpro Guideline Development Tool. McMaster University, 2020 (developed by Evidence Prime, Inc.). Available from gradepro.org.] to report the certainty of evidence. Disagreements between the two review authors over the quality assessment of the evidence were resolved by discussion, with the involvement of the third author (A.N.) whenever necessary.

Based on the Grade approach, the quality of evidence is assessed based on the risk of bias, inconsistency, indirectness, imprecision, or publication bias. Quality of evidence is rated as:

- High certainty: We are very confident that the true effect lies close to that of the estimate of the effect.
- Moderate certainty: We are moderately confident in the effect estimate. The true effect is likely to be close to the estimate of the effect, but there is a possibility that it is substantially different.
- Low certainty: Our confidence in the effect estimate is limited. The true effect may be substantially different from the estimate of the effect.
- Very low certainty: We have very little confidence in the effect estimate. The true effect is likely to be substantially different from the estimate of effect^40^.

## Results

### Study selection

**Figure 1:**
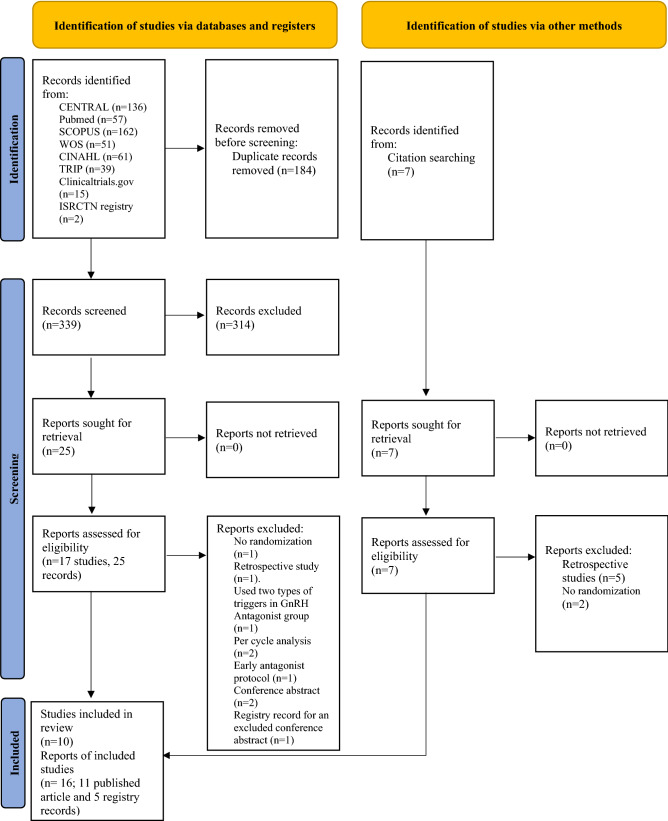
Flow diagram selection process.

The database search returned a total of 523 records (CENTRAL n = 136, Pubmed n = 57, SCOPUS n = 162, Web of Science n = 51, CINAHL n = 61, TRIP n = 39, Clinicaltrials.gov n = 15, ISRCTN registry n = 2). Additionally, seven records were identified from the hand searches, but none of them met our inclusion criteria. After duplicates removal (n = 184), 339 records were left to be screened, of which 314 were excluded based on the title and abstract. The full-text review of the remaining 25 records (17 studies) led to a rejection of further nine records (7 studies). We only included Conventional GnRH antagonist protocols. Thus, we excluded the Early GnRH antagonist arm from 3 included studies. The excluded studies/arms are shown in Table 1 with reasons for exclusion. A remaining total of 16 records, 11 published article and five registry records (10 studies) met the inclusion criteria and were included in the meta-analysis. (See Fig. 1 for details of this process). The characteristics of included RCTs are summarized in Supplementary Table S4. The included studies randomized a total of 1214 women.

**Table 1:** Excluded studies.

Databases search exclusions:	
Study ID	Reasons for exclusion
Ashrafi et al.^41^	Used two types of triggers in the GnRH antagonist group; hCG trigger in general and GnRH agonist trigger in cases at high risk of OHSS
Chen et al.^42^	A retrospective study
Choi et al. ^43^ (one arm excluded)	A three-arm study included; an Early Flexible GnRH antagonist protocol, a Conventional Flexible GnRH antagonist protocol, and a Long GnRH agonist protocol. We only included the Conventional Flexible GnRH antagonist arm and the Long GnRH agonist arm
Choi et al. ^44,45^	Used a Per cycle analysis. Yet, we were not capable of reaching the study corresponding author due to an invalid e-mail
Lainas et al.^25^	Used an Early GnRH antagonist protocol in the GnRH antagonist arm
Mokhtar et al.^23,46^ (one arm excluded)	A three-arm study included; an Early Flexible GnRH antagonist protocol, a Conventional Flexible GnRH antagonist protocol, and a Long GnRH agonist protocol. We only included the Conventional Flexible GnRH antagonist arm and the Long GnRH agonist arm
Moshin et al.^47^	A conference abstract. The per-cycle analysis was most likely used in this study. Yet, no response from the author was obtained to confirm its eligibility
Shin et al.^27,48^ (one arm excluded)	A three-arm study included; an Early Fixed GnRH antagonist protocol, a Conventional Fixed GnRH antagonist protocol and a Long GnRH agonist protocol. We only included the Conventional Fixed GnRH antagonist arm and the Long GnRH agonist arm
Vrtačnik-Bokal et al.^49^	Not a randomized controlled trial
Zeinalzadeh et al. ^50,51^	A conference abstract with no numerical data, Yet, no response from the author was obtained to provide the study results
**Hand search exclusions:**	
Study ID	Reasons for exclusion
Orvieto et al.^52^	A retrospective case series
Orvieto et al.^53^	A retrospective case series
Kaur et al.^54^	Not a randomized controlled trial
Kdous et al.^55^	A retrospective case–control study
Kdous et al.^56^	A retrospective case–control study
Onofriescu et al.^57^	Not a randomized controlled trial
Segal et al.^58^	A retrospective case series

**GnRH**: Gonadotropin-releasing hormone, **hCG**: Human chorionic gonadotropin, **OHSS**: ovarian hyperstimulation syndrome.

### Study characteristics

Nine studies were single-center RCTs^17,18,23,43,59–63^, while one study was a multi-center RCT^27^. All of the included trials had a parallel study design. Inclusion and exclusion criteria were available in all studies. The included studies were published between 2005 and 2018, and were conducted in Greece^62^, Iran^17,23,60^, Korea^27,43^, Poland^61^, Serbia^63^ and Turkey^18,59^. The number of randomized women in the GnRH antagonist arms ranged from 14 to 150 women, while in the GnRH agonist arms from 13 to 150 women. All studies initiated an oral contraceptive pill pre-treatment (OCPs) for all participants.

All included studies compared GnRH antagonist protocols with the Long GnRH agonist protocol. The Flexible GnRH antagonist protocol was used in 8 studies^17,23,43,59–63^, while the Fixed GnRH antagonist protocol was used in 2 studies^18,27^. The used GnRH antagonists were Cetrorelix in 8 studies RCTs^17,23,27,43,59–61,63^, and Ganirelix in 2 studies^18,62^, while used GnRH agonists were Buserelin in 3 studies^17,23,60^, Leuprolide in 2 studies^18,59^, Triptorelin in 5 studies^27,43,61–63^. The gonadotropin starting dose varied from 150 to 225 IUs of recombinant follicle-stimulating hormone (r-FSH) or human menopausal gonadotropin (hMG) in the GnRH antagonist group while 150 to 300 IUs in the GnRH agonist group. However, only one study did not report gonadotropin starting dose^43^. All included studies used hCG trigger. All the included studies transferred embryos during the cleavage stage (D2/D3) to the participants except for one studies^27^ which transferred both cleave stage embryos and blastocyst embryos. Two studies performed intention-to-treat analysis (ITT)^60,62^, 3 studies performed modified ITT (mITT; ITT with excluding missing outcome data)^17,27,59^, 4 studies performed ITT/mITT based on the outcome^18,23,43,63^ and one study performed a per-protocol analysis^61^. Seven studies reported OHSS diagnosis criteria^17,18,23,27,60,62,63^, while only three studies reported cycle cancellation criteria^17,18,62^. For more details about the studies characteristics, see Supplementary Table S4.

### Risk of bias of the included studies

Regarding bias arising from the randomization process, four studies^17,18,27,61^ were at “low risk” of bias, five studies^23,43,59,62,63^ held “some concern”, and one study was at “high risk” of bias^60^. Randomization was accomplished using a random numbers table in two studies^18,59^, a computer-generated random number table in four studies^23,61–63^ and a web-based system in one study^27^. However, the remaining studies did not provide any information regarding the methods were used for sequence generation. Allocation concealment was suitably performed using sealed opaque envelopes in 3 studies^17,18,61^ and using a web-based system in one study^27^. Nevertheless, the remaining studies did not provide any information about the possibility of allocation concealment or what methods were used to conceal it. Baseline imbalance arises from the randomization process was suspected in one study^60^ since there was a significant difference in the means of age between the two groups; with younger patients in the GnRH antagonist one, and no information about the randomization process or allocation concealment were provided. However, since ROB 2.0 tool assesses the risk of bias in the remaining domains at the outcome level, the information about the risk of bias regarding the other domains are reported separately for each outcome.

### Live birth rate per randomized woman (LBR)

**Figure 2:**
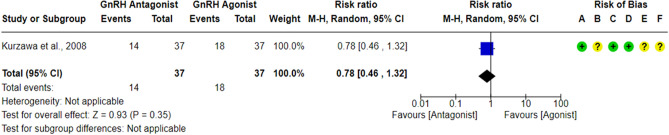
Forest Plot: Live Birth Rate Per Randomized Woman. (**A**) Bias arising from the randomization process; (**B**) Bias due to deviations from intended interventions; (**C**) Bias due to missing outcome data;(**D**) Bias in measurement of the outcome; (**E**) Bias in selection of the reported result and (**F**) overall bias.

Only one RCT^61^ compared the LBR between the GnRH antagonist and GnRH agonist protocols in 74 PCOS women, and it did not report any significant differences in LBR between the GnRH antagonist protocol and the Long GnRH agonist protocol (RR = 0.78, 95% CI: [0.46 to 1.32]; *P* = 0.35; low-quality evidence; Fig. 2). The study was judged to be at “low risk” of bias due to missing outcome data and bias in outcome measurement, taking into account the number of missing outcome data and the event risk. Furthermore, it is an objective outcome, and its assessment would not be influenced by knowledge of the intervention received. However, it held “some concern” regarding the risk of bias due to deviations from intended interventions since a per-protocol analysis was conducted. Yet, the number of excluded participants was unlikely to have a substantial impact on the results. The study had a registry record, and the outcome was specified in it, but it was retrospectively registered. Using GRADE, we judged the certainty of evidence as low; we down-graded it by two levels due to concerns over imprecision.

### Ongoing pregnancy rate per randomized woman (OPR)

**Figure 3:**
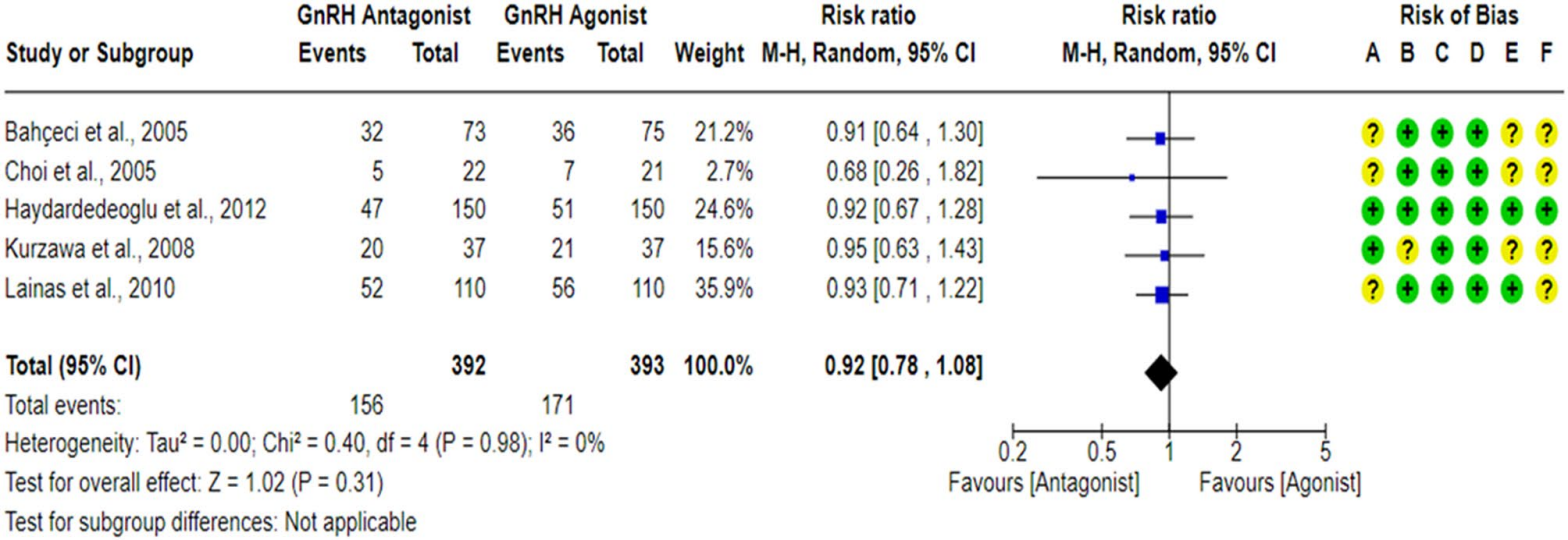
Forest Plot: Ongoing Pregnancy Rate Per Randomized Woman. (**A**) Bias arising from the randomization process; (**B**) Bias due to deviations from intended interventions; (**C**) Bias due to missing outcome data;(**D**) Bias in measurement of the outcome; (**E**) Bias in selection of the reported result and (**F**) overall bias.

A total of five RCTs^18,43,59,61,62^ compared the OPR between the GnRH antagonist and GnRH agonist protocols in 785 PCOS women. All included studies were at “low risk” of bias due to deviations from intended interventions except for one study^61^, which held “some concern” since a per-protocol analysis was conducted. Yet, the number of excluded participants was unlikely to have a substantial impact on the results. All included studies were at “low risk” of bias due to missing outcome data and bias in outcome measurement, taking into account the number of missing outcome data, the causes of missingness, and the event risk. Furthermore, it is an objective outcome, and its assessment would not be influenced by knowledge of the intervention received. Only two studies^18,62^ had a prospective registry record, and the outcome was pre-specified in it, so they were at “low risk” of bias in selection of the reported result. Pooling the results of these RCTs did not show any significant differences in OPR between the GnRH antagonist protocols and the Long GnRH agonist protocol (RR = 0.92, 95% CI: [0.78 to 1.08]; *P* = 0.31; I^2^ = 0%; χ^2^-*P* = 0.98; high-quality evidence; Fig. 3).

### OHSS rate per randomized woman

**Figure 4:**
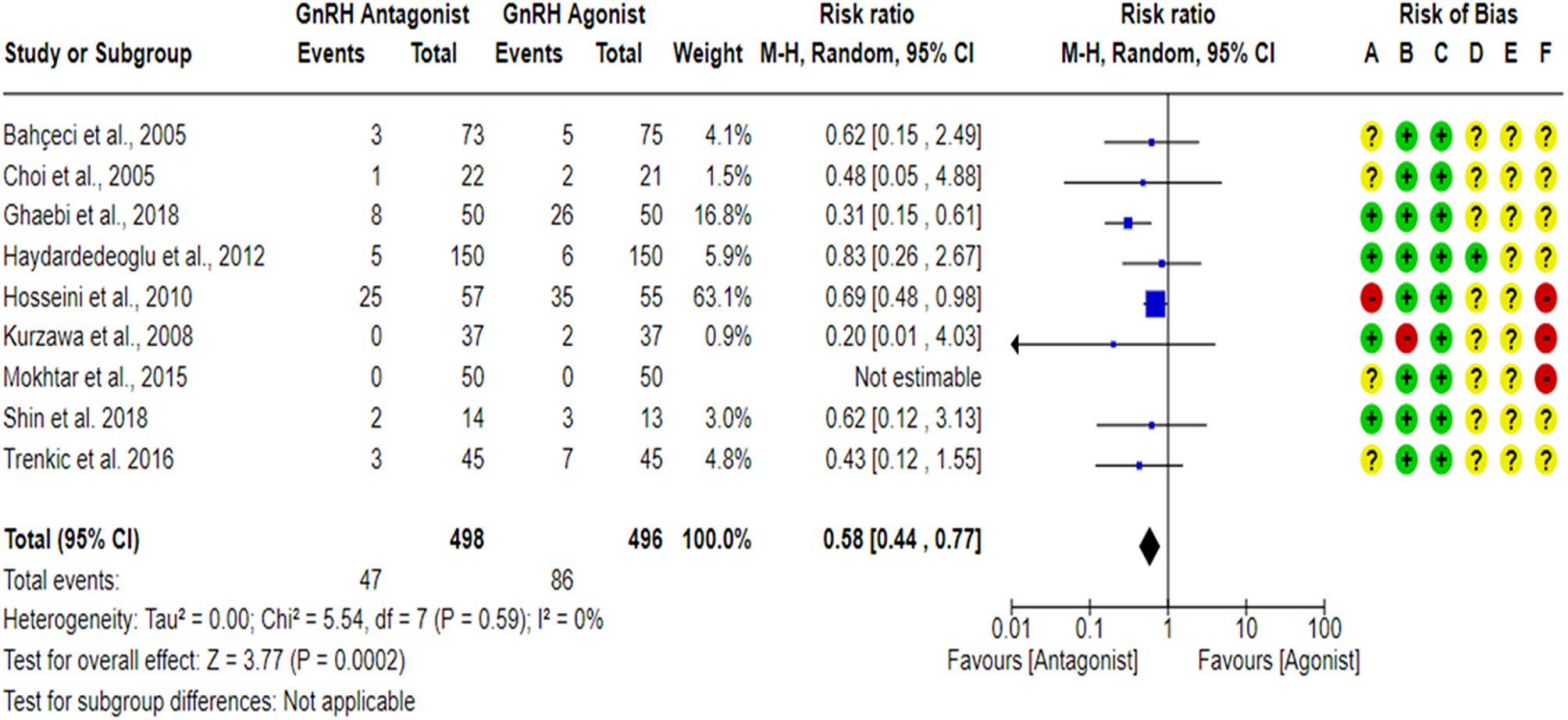
Forest Plot: OHSS Rate Per Randomized Woman. (**A**) Bias arising from the randomization process; (**B**) Bias due to deviations from intended interventions; (**C**) Bias due to missing outcome data;(**D**) Bias in measurement of the outcome; (**E**) Bias in selection of the reported result and (**F**) overall bias.

**Figure 5:**
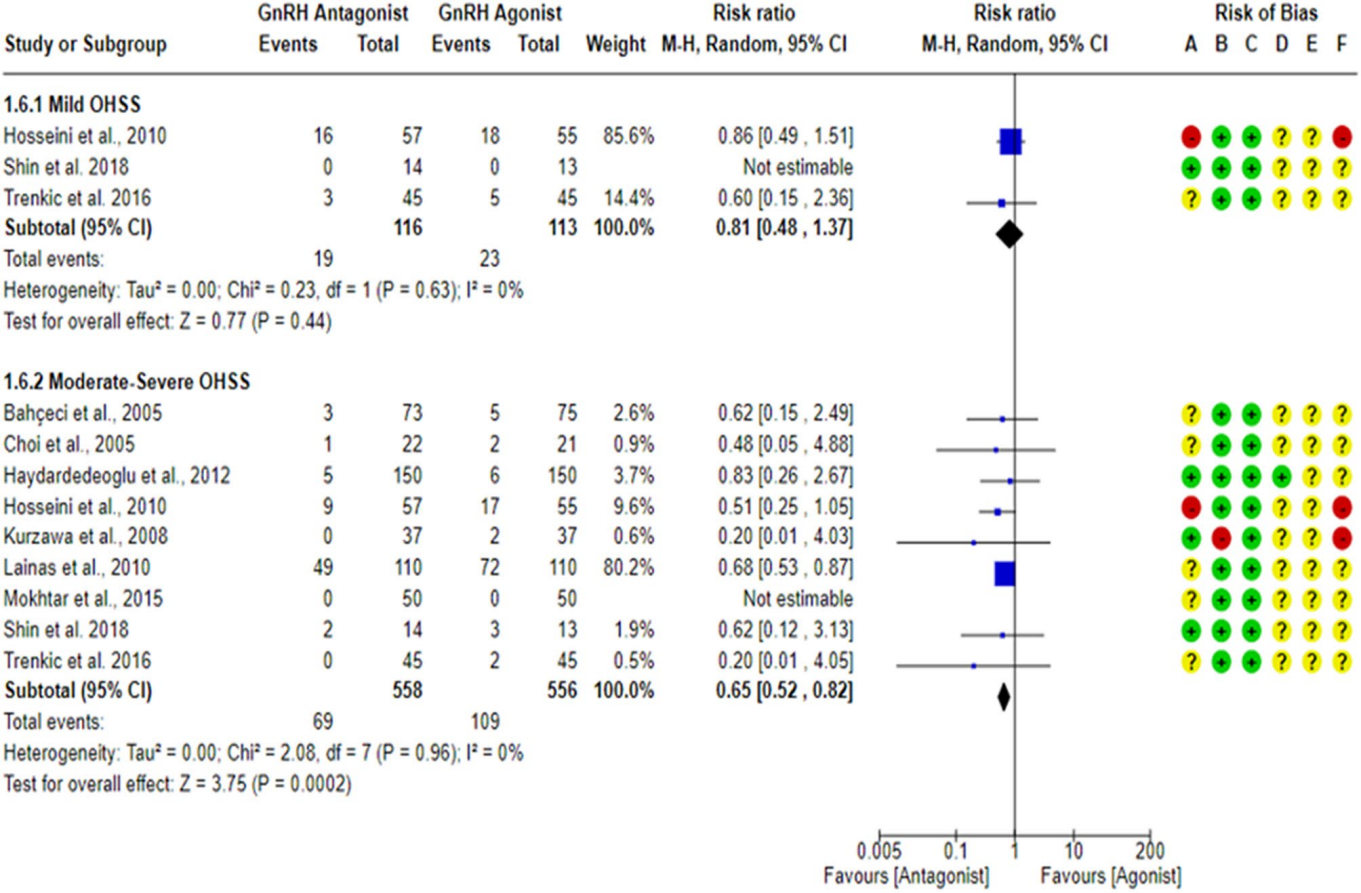
Forest Plot: OHSS Rate Per Randomized Woman (Per OHSS Grade). (**A**) Bias arising from the randomization process; (**B**) Bias due to deviations from intended interventions; (**C**) Bias due to missing outcome data;(**D**) Bias in measurement of the outcome; (**E**) Bias in selection of the reported result and (**F**) overall bias.

All included studies^17,18,23,27,43,59–63^ compared the OHSS rate between the GnRH antagonist and GnRH agonist protocols. All included studies were at “low risk” of bias due to missing outcome data and bias due to deviations from intended interventions except for one study^61^, which was at “high risk” of bias due to deviations from intended interventions since a per-protocol analysis was conducted, and the number of excluded participants was likely to have a substantial impact on the results. One study^18^ was at “low risk” of bias in outcome measurement since the investigators were blinded (based on the information provided in the registry record), while nine studies^17,23,27,43,59–63^ held “some concern” of bias due to failure to blind the outcome’ assessors or not reporting whether the outcome assessors were blinded or not. Moreover, OHSS is considered a subjective outcome as it is usually diagnosed based on particular criteria, so we have some concerns about potential bias. Only two studies^18,62^ had a prospective registry record, and the outcome was pre-specified in it. However, they did not report the outcome measurement time-point. In addition, one study^62^ reported the OHSS rate based on its grade, with grade I representing “none or mild OHSS”, so we were incapable of determining the overall number of OHSS cases, but we could determine the number of Moderate-Severe cases. Thus, only 9 RCTs^17,18,23,27,43,59–61,63^ (994 PCOS women) were included in the meta-analysis concerning the overall OHSS rate. Pooling the results of these RCTs showed a significant lower OHSS rate when women were treated with the GnRH antagonist protocols compared to those treated with the Long GnRH agonist protocol (RR = 0.58, 95% CI: [0.44 to 0.77]; *P* = 0.0002; NNTB 14, NNTB 95% CI: [11 to 26]; I^2^ = 0%; χ^2^-P = 0.59; low-quality evidence; Fig. 4). Based on the results of the sensitivity analysis, the results were robust against the exclusion of high risk of bias studies^60,61^ (RR = 0.44, 95% CI: [0.28 to 0.71]; *P* = 0.0007; I^2^ = 0%; χ^2^-*P* = 0.76). Using GRADE, we judged the certainty of evidence as low; we down-graded it by two levels, one level due to concerns over imprecision and one due to concerns over the risk of bias. We also conducted a subgroup analysis based on the degree of OHSS [Mild VS Moderate-Severe]. Based on the result of the subgroup analysis, there was not any significant difference in Mild OHSS rate between the GnRH antagonist protocols and the Long GnRH agonist protocol (3 studies; 229 women; RR = 0.81, 95% CI: [0.48 to 1.37]; *P* = 0.44; I^2^ = 0%; χ^2^-*P* = 0.63; very-low quality evidence; Fig. 5). Using GRADE, we judged the certainty of evidence as very low; we down-graded it by three levels, two levels due to concerns over imprecision and one due to concerns over the risk of bias. On the other hand, a significant lower Moderate-Severe OHSS rate was noticed when women were treated with GnRH antagonist protocols compared to those treated with the Long GnRH agonist protocol (9 studies; 1114 women; RR = 0.65, 95% CI: [0.52 to 0.82]; *P* = 0.0002; NNTB 15, NNTB 95% CI: [11 to 29]; I^2^ = 0%; χ^2^-*P* = 0.96; very-low quality evidence; Fig. 5). Using GRADE, we judged the certainty of evidence as very low; we down-graded it by three levels, two levels due to concerns over imprecision and one due to concerns over the risk of bias.

### Clinical pregnancy rate per randomized woman (CPR)

**Figure 6:**
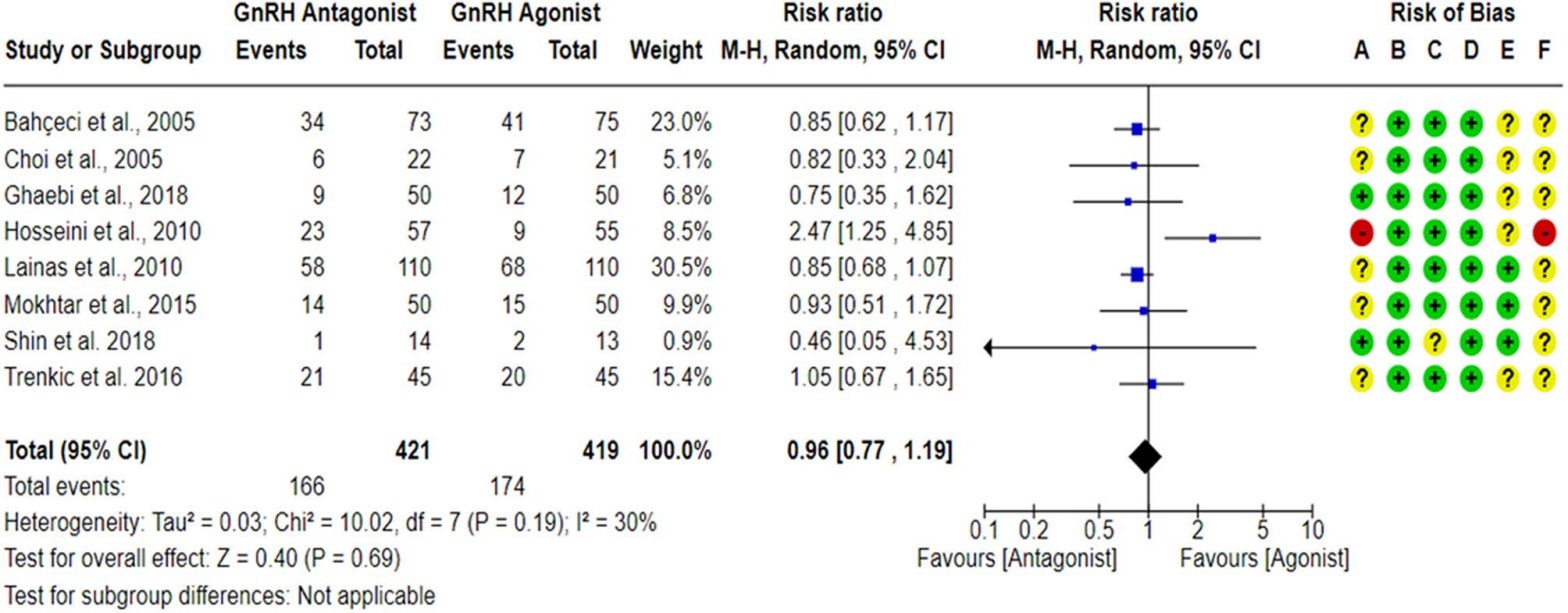
Forest Plot: Clinical Pregnancy Rate Per Randomized Woman. (**A**) Bias arising from the randomization process; (**B**) Bias due to deviations from intended interventions; (**C**) Bias due to missing outcome data;(**D**) Bias in measurement of the outcome; (**E**) Bias in selection of the reported result and (**F**) overall bias.

A total of eight RCTs^17,23,27,43,59,60,62,63^ compared the CPR between the GnRH antagonist and GnRH agonist protocols in 840 PCOS women. All included studies were judged to be at “low risk” of bias due to deviations from intended interventions and bias in outcome measurement taking into account the type of analysis. In addition, CPR is an objective outcome, and its assessment would not be influenced by knowledge of the intervention received. All included studies were at “low risk” of bias due to missing outcome data except for one study^27^, which held “some concern” based on the number of missing outcome data, the causes of missingness, and the event risk. Only three studies^23,27,62^ had a prospective registry record, and the outcome was pre-specified in it, so they were at “low risk” of bias in selection of the reported result. Pooling the results of these RCTs did not show any significant differences in CPR between the GnRH antagonist protocols and the Long GnRH agonist protocol (RR = 0.96, 95% CI: [0.77 to 1.19]; *P* = 0.69; I^2^ = 30%; χ^2^-*P* = 0.19; high-quality evidence; Fig. 6). Based on the results of the sensitivity analysis, the results were robust against the exclusion of the high risk of bias study^60^ (RR = 0.87, 95% CI: [0.74 to 1.02]; *P* = 0.09; I^2^ = 0%; χ^2^-*P* = 0.98).

### Multiple pregnancy rate per randomized woman (MPR)

**Figure 7:**
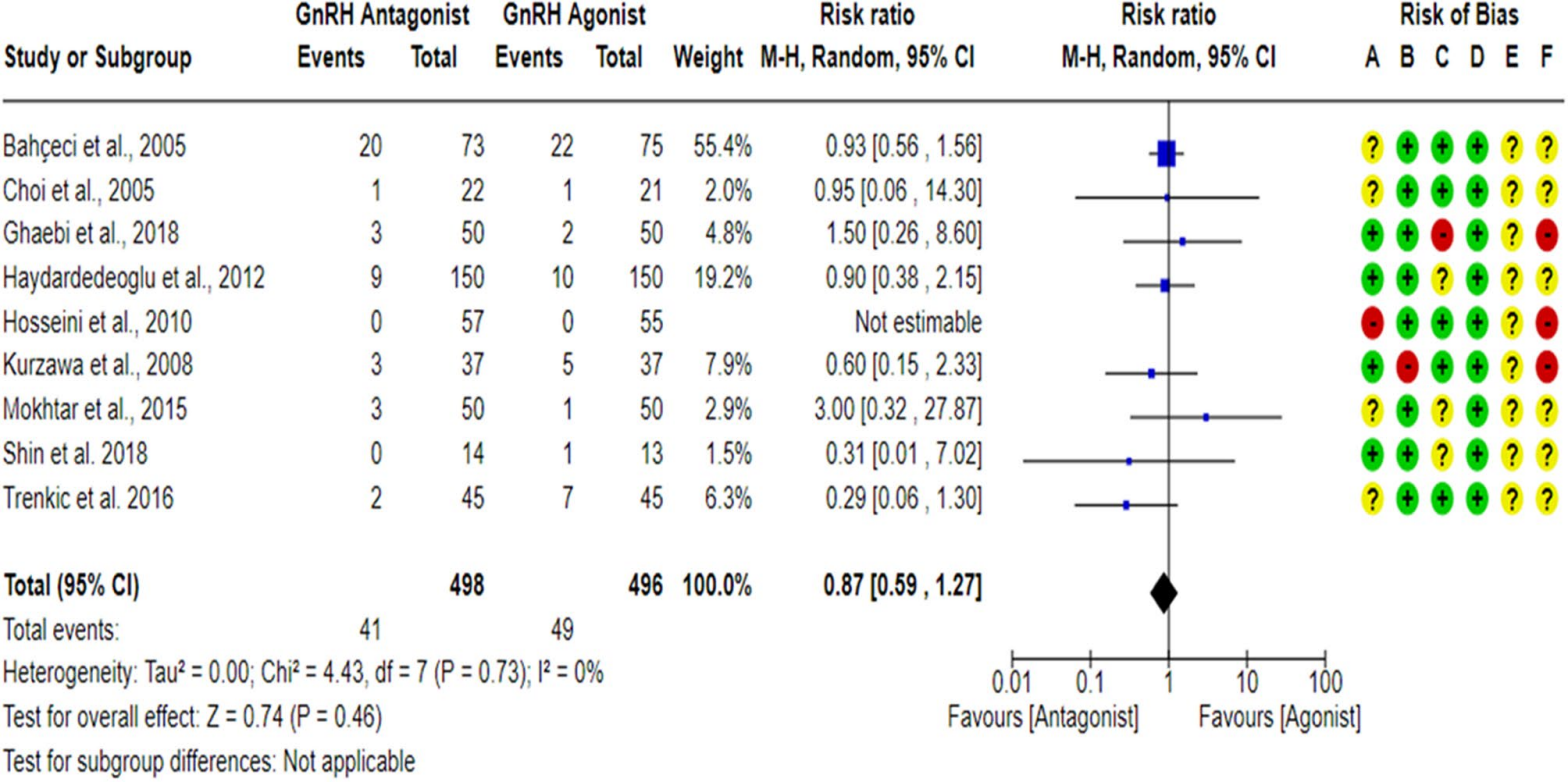
Forest Plot: Multiple Pregnancy Rate Per Randomized Woman. (**A**) Bias arising from the randomization process; (**B**) Bias due to deviations from intended interventions; (**C**) Bias due to missing outcome data;(**D**) Bias in measurement of the outcome; (**E**) Bias in selection of the reported result and (**F**) overall bias.

A total of nine^17,18,23,27,43,59–61,63^ RCTs compared the MPR between the GnRH antagonist and GnRH agonist protocols in 994 PCOS women. All included studies were at “low risk” of bias due to deviations from intended interventions except for one study^61^, which was at “high risk” of bias since a per-protocol analysis was conducted, and the number of excluded participants was likely to have a substantial impact on the results. All included studies were at “low risk” of bias due to missing outcome data except for three studies^18,23,27^, which held “some concern”, and one study^17^ was at “high risk” of bias based on the number of missing data, the causes of missingness, and the event risk. However, we judged the latter as “high risk” of bias since drop-out cases were only observed in the GnRH antagonist group, and the reasons for dropping out were not reported. All included studies were at “low risk” of bias in outcome measurement since it is an objective outcome, and its assessment would not be influenced by knowledge of the intervention received. Pooling the results of these RCTs did not show any significant differences in MPR between the GnRH antagonist protocols and the Long GnRH agonist protocol (RR = 0.87, 95% CI: [0.59 to 1.27]; *P* = 0.46; I^2^ = 0%; χ^2^-*P* = 0.73; very-low quality evidence; Fig. 7). Based on the results of the sensitivity analysis, the results were robust against the exclusion of high risk of bias studies^17,60,61^ (RR = 0.87, 95% CI: [0.58 to 1.31]; *P* = 0.50; I^2^ = 0%; χ^2^-*P* = 0.58). Using GRADE, we judged the certainty of evidence as very low; we down-graded it by three levels, two levels due to concerns over imprecision and one due to concerns over the risk of bias.

### Miscarriage rate per randomized woman (MR)

**Figure 8:**
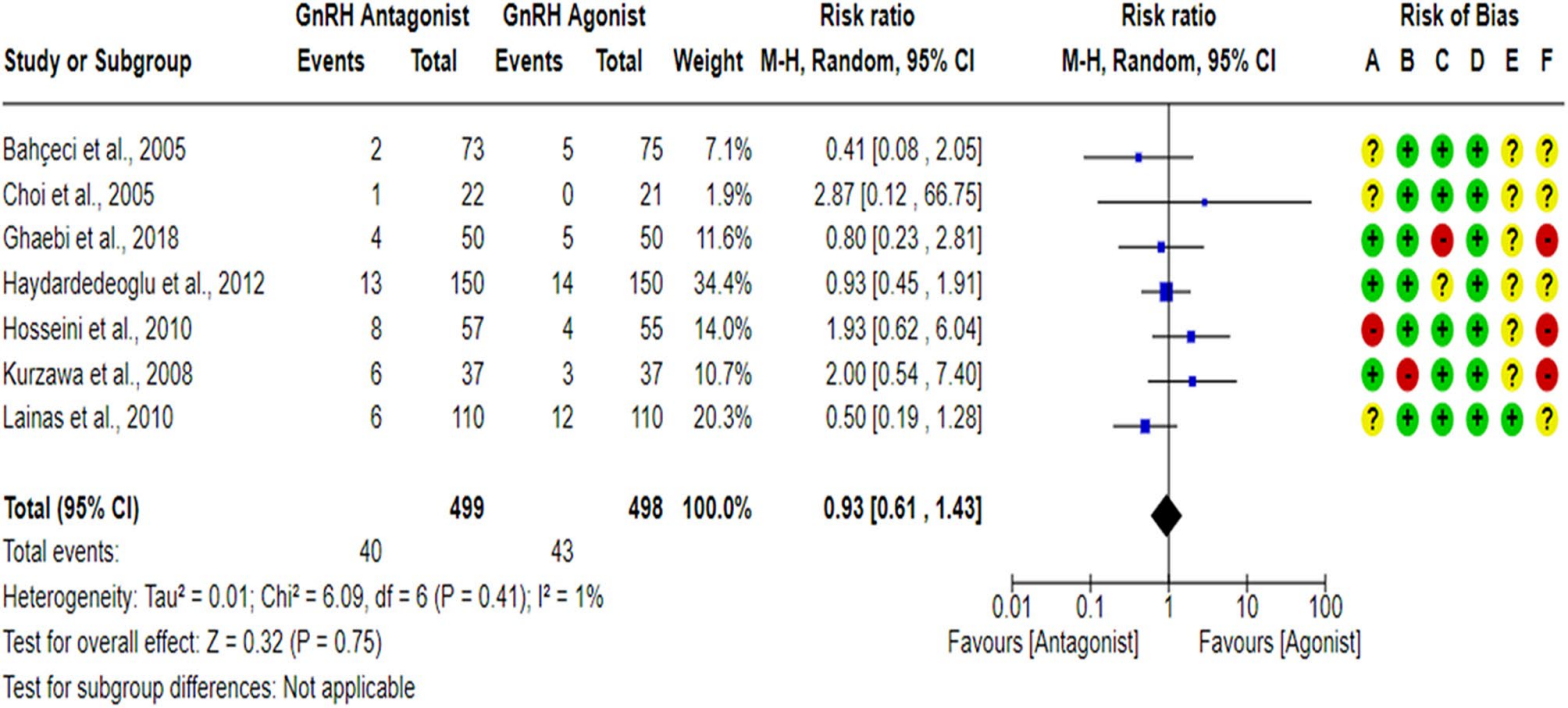
Forest Plot: Miscarriage Rate Per Randomized Woman. (**A**) Bias arising from the randomization process; (**B**) Bias due to deviations from intended interventions; (**C**) Bias due to missing outcome data;(**D**) Bias in measurement of the outcome; (**E**) Bias in selection of the reported result and (**F**) overall bias.

A total of seven RCTs^17,18,43,59–62^ compared the MR between the GnRH antagonist and GnRH agonist protocols in 997 PCOS women. All included studies were at “low risk” of bias due to deviations from intended interventions except for one study^61^, which was at “high risk” of bias since a per-protocol analysis was conducted, and the number of excluded participants was likely to have a substantial impact on the results. All included studies were at “low risk” of bias due to missing outcome data except for one study^18^, which held “some concern”, and one study^17^ was at “high risk” of bias based on the number of missing outcome data, the causes of missingness, and the event risk. However, we judged the latter as “high risk” of bias since drop-out cases were only observed in the GnRH antagonist group, and the reasons for dropping out were not reported. All included studies were at “low risk” of bias in outcome measurement since it is an objective outcome, and its assessment would not be influenced by knowledge of the intervention received. Pooling the results of these RCTs did not show any significant differences in MR between the GnRH antagonist protocols and the Long GnRH agonist protocol (RR = 0.93, 95% CI: [0.61 to 1.43]; *P* = 0.75; I^2^ = 1%; χ^2^-*P* = 0.41; very-low quality evidence; Fig. 8). Based on the results of the sensitivity analysis, the results were robust against the exclusion of high risk of bias studies^17,60,61^ (RR = 0.72, 95% CI: [0.42 to 1.23]; *P* = 0.23; I^2^ = 0%; χ^2^-*P* = 0.52). Using GRADE, we judged the certainty of evidence as very low; we down-graded it by three levels, two levels due to concerns over imprecision and one due to concerns over the risk of bias.

### Cycle cancellation rate per randomized woman (CCR)

**Figure 9:**
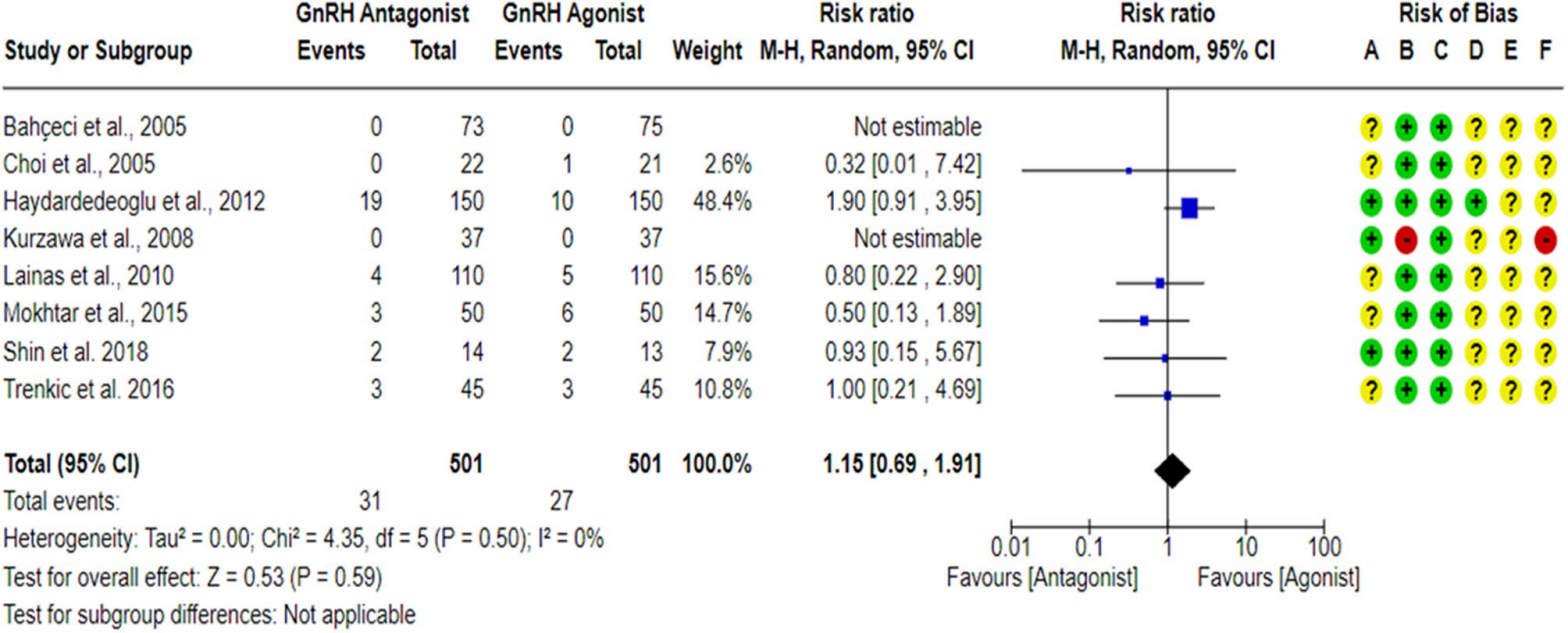
Forest Plot: Cycle Cancellation Rate Per Randomized Woman. (**A**) Bias arising from the randomization process; (**B**) Bias due to deviations from intended interventions; (**C**) Bias due to missing outcome data;(**D**) Bias in measurement of the outcome; (**E**) Bias in selection of the reported result and (**F**) overall bias.

**Figure 10:**
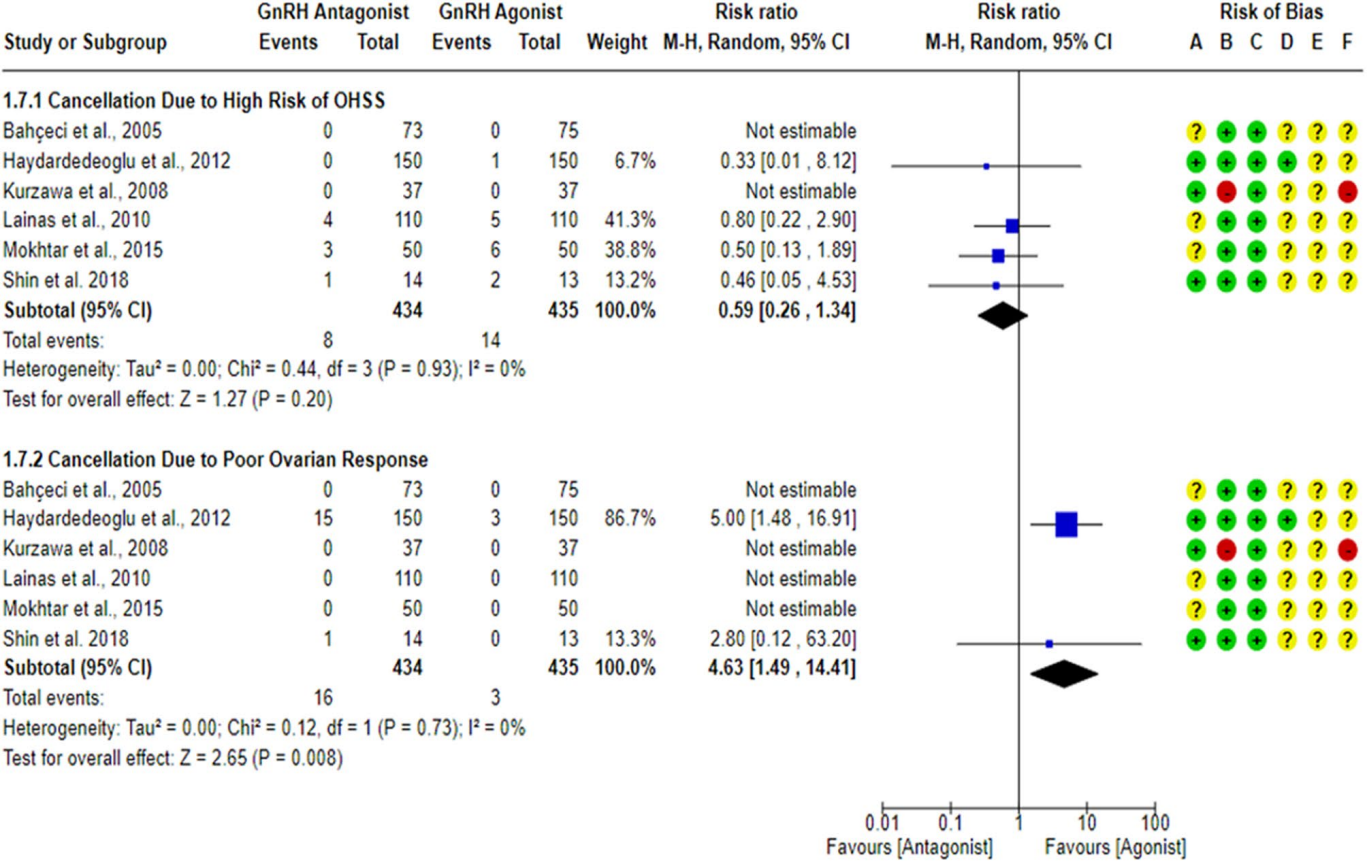
Forest Plot: Cycle Cancellation Rate Per Randomized Woman (Per Cause of Cancellation). (**A**) Bias arising from the randomization process; (**B**) Bias due to deviations from intended interventions; (**C**) Bias due to missing outcome data;(**D**) Bias in measurement of the outcome; (**E**) Bias in selection of the reported result and (**F**) overall bias.

A total of eight studies^18,23,27,43,59,61–63^ reported cycle cancellation rate between the GnRH antagonist and GnRH agonist protocols in 1002 PCOS women. Although one study^17^ reported cycle cancellation criteria, it was unclear if any cycle cancellation cases would have been noted in the study’s groups. Thus, this study^17^ was not included in the meta-analysis. All included studies were at “low risk” of bias due to missing outcome data and bias due to deviations from intended interventions except for one study^61^, which was at “high risk” of bias due to deviations from intended interventions even though no cycle cancellation cases were reported, but a per-protocol analysis was conducted, and the number of excluded participants was likely to have a substantial impact on the results. One study^18^ was at “low risk” of bias in outcome measurement since the investigators were blinded (based on the information provided in the registry record), while seven studies^23,27,43,59,61–63^ held “some concern” of bias due to failure to blind the outcome’ assessors or not being clear in reporting whether the outcome assessors were blinded or not. Cycle cancellation is considered a subjective outcome since the cancellation decision is usually taken based on particular criteria, so we have some concerns about potential bias. Pooling the results of these RCTs did not show any significant differences in CCR between the GnRH antagonist protocols and the Long GnRH agonist protocol (RR = 1.15, 95% CI: [0.69 to 1.91]; *P* = 0.59; I^2^ = 0%; χ^2^-*P* = 0.50; very-low quality evidence; Fig. 9). Based on the results of the sensitivity analysis, the results were robust against the exclusion of the high risk of bias study^61^ (RR = 1.15, 95% CI: [0.69 to 1.91]; *P* = 0.59; I^2^ = 0%; χ^2^-*P* = 0.50). Using GRADE, we judged the certainty of evidence as very low; we down-graded it by three levels, two levels due to concerns over imprecision and one due to concerns over the risk of bias. We also conducted a subgroup analysis based on the causes of cycle cancellation [High Risk of OHSS VS Poor Ovarian Response]. Two studies^43,63^ did not report the causes of cycle cancellation, so they were excluded from the subgroup analysis. Based on the result of the subgroup analysis, there was not any significant difference in the rate of cycle cancellation due to High Risk of OHSS between the GnRH antagonist protocols and the Long GnRH agonist protocol (6 studies; 869 women; RR = 0.59, 95% CI: [0.26 to 1.34]; *P* = 0.20; I^2^ = 0%; χ^2^-*P* = 0.93; very-low quality evidence; Fig. 10). Using GRADE, we judged the certainty of evidence as very low; we down-graded it by three levels, two levels due to concerns over imprecision and one due to concerns over the risk of bias. On the other hand, a significant increase in cycle cancellation rate due to Poor Ovarian Response was noticed when women were treated with GnRH antagonist protocol compared to those treated with the Long GnRH agonist protocol (6 studies; 869 women; RR = 4.63, 95% CI: [1.49 to 14.41]; *P* = 0.008; NNTH 40, NNTH 95% CI: [11 to 292]; I^2^ = 0%; χ^2^-*P* = 0.73; very-low quality evidence; Fig. 10). Using GRADE, we judged the certainty of evidence as very low; we down-graded it by three levels, two levels due to concerns over imprecision and one due to concerns over the risk of bias.

### Stimulation duration

**Figure 11:**
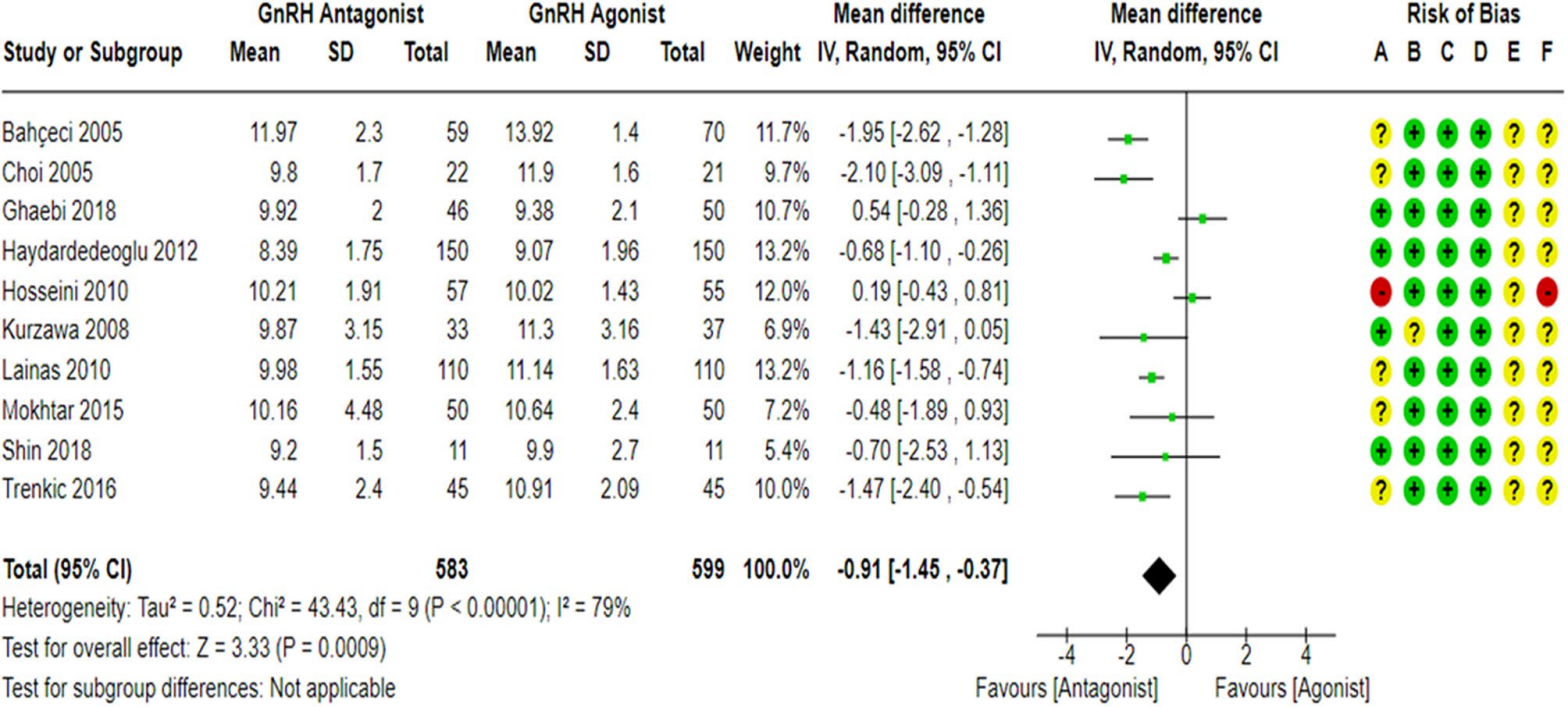
Forest Plot: Stimulation Duration. (**A**) Bias arising from the randomization process; (**B**) Bias due to deviations from intended interventions; (**C**) Bias due to missing outcome data;(**D**) Bias in measurement of the outcome; (**E**) Bias in selection of the reported result and (**F**) overall bias.

All the included studies^17,18,23,27,43,59–63^ compared the stimulation duration between the GnRH antagonist and GnRH agonist protocols in 1182 PCOS women. All included studies were at “low risk” of bias due to deviations from intended interventions except for one study^61^, which held “some concern” since a per-protocol analysis was conducted. Yet, the number of excluded participants was unlikely to have a substantial impact on the results. All included studies were at “low risk” of bias due to missing outcome data and bias in outcome measurement, taking into account the number of missing outcome data, and the causes of missingness. In addition, it is an objective outcome, and its assessment would not be influenced by knowledge of the intervention received. Pooling the results of these RCTs showed that the stimulation duration was significantly shorter in the GnRH antagonist protocols compared with the Long GnRH agonist protocol (WMD = − 0.91, 95% CI: [− 1.45 to − 0.37] day; *P* = 0.0009; I^2^ = 79%; χ^2^-*P* < 0.00001; very low-quality evidence; Fig. 11). Based on the results of the sensitivity analysis, the results were robust against the exclusion of the high risk of bias study^60^ (WMD = − 1.06, 95% CI: [− 1.59 to − 0.54] day; *P* < 0.0001; I^2^ = 74%; χ^2^-P = 0.0002) and the removal of estimated outcome data^61,62^ (WMD = − 0.83, 95% CI: [− 1.51 to − 0.14] day; *P* = 0.02; I^2^ = 83%; χ^2^-*P* < 0.00001). Subgroup analyses were performed to detect potential sources of heterogeneity based on the type of GnRH antagonist protocol used (Flexible VS Fixed). The results of subgroup analysis showed that the stimulation duration was significantly shorter in the GnRH antagonist protocol compared with the Long GnRH agonist protocol either when using Flexible GnRH antagonist protocol (8 RCTs; 860 participants; WMD = − 0.97, 95% CI: [− 1.66 to − 0.28] day; *P* = 0.006; I^2^ = 83%; χ^2^-*P* < 0.00001) or Fixed GnRH antagonist protocol (2 RCTs; 322 participants; WMD = − 0.68, 95% CI: [-1.09 to − 0.27] day; *P* = 0.001; I^2^ = 0%; χ^2^-*P* = 0.98) and the result of the subgroup analysis was not significant (χ^2^-*P* = 0.49, I^2^ = 0%). Using GRADE, we judged the certainty of evidence as very low; we down-graded it by three levels, two levels due to concerns over heterogeneity and one over imprecision.

### Gonadotropin dose

**Figure 12:**
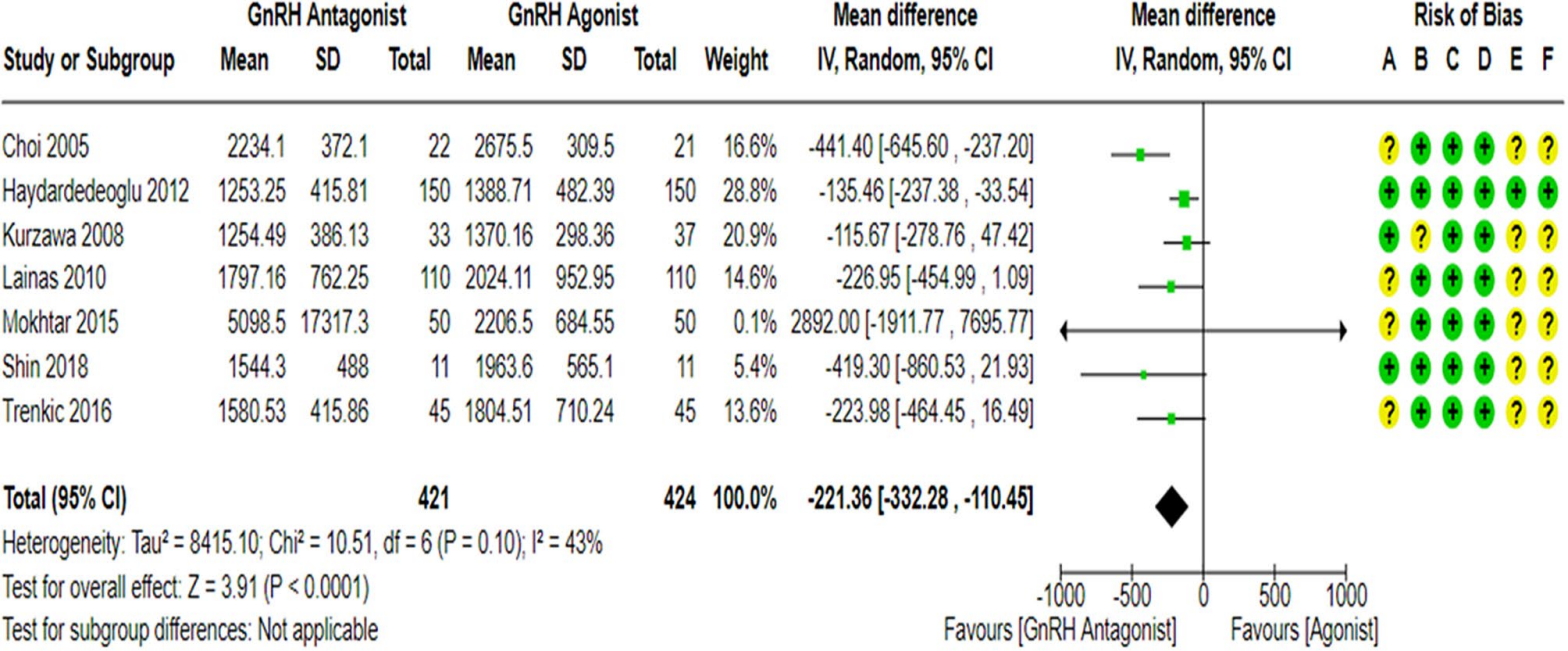
Forest Plot: Gonadotropin Dose. (A) Bias arising from the randomization process; (**B**) Bias due to deviations from intended interventions; (**C**) Bias due to missing outcome data;(**D**) Bias in measurement of the outcome; (**E**) Bias in selection of the reported result and (**F**) overall bias.

Seven RCTs^18,23,27,43,61–63^ compared gonadotropin consumption (IUs) during COS between the GnRH antagonist and GnRH agonist protocols in 845 PCOS women. All of them used r-FSH for ovarian stimulation. All included studies were at “low risk” of bias due to deviations from intended interventions except for one study^61^, which held “some concern” since a per-protocol analysis was conducted. However, the number of excluded participants was unlikely to have a substantial impact on the results. All included studies were at “low risk” of bias due to missing outcome data and bias in outcome measurement, taking into account the number of missing outcome data, and the causes of missingness. Moreover, it is an objective outcome, and its assessment would not be influenced by knowledge of the intervention received. Only one study^18^ had a prospective registry record, and the outcome was pre-specified in it, so they were at “low risk” of bias in selection of the reported result. Pooling the results of these RCTs showed that the gonadotropin consumption (IUs) during COS was significantly lower in the GnRH antagonist protocols compared with the Long GnRH agonist protocol (WMD = − 221.36, 95% CI: [− 332.28 to − 110.45] IUs; *P* < 0.0001; I^2^ = 43%; χ^2^-*P* = 0.1; high-quality evidence; Fig. 12). Based on the results of the sensitivity analysis, the results were robust against the removal of estimated outcome data^61,62^ (WMD = − 269.12, 95% CI: [− 448.23 to − 0.90] IU; *P* = 0.003; I^2^ = 58%; χ^2^-*P* = 0.05).

### E2 levels on hCG day

**Figure 13:**
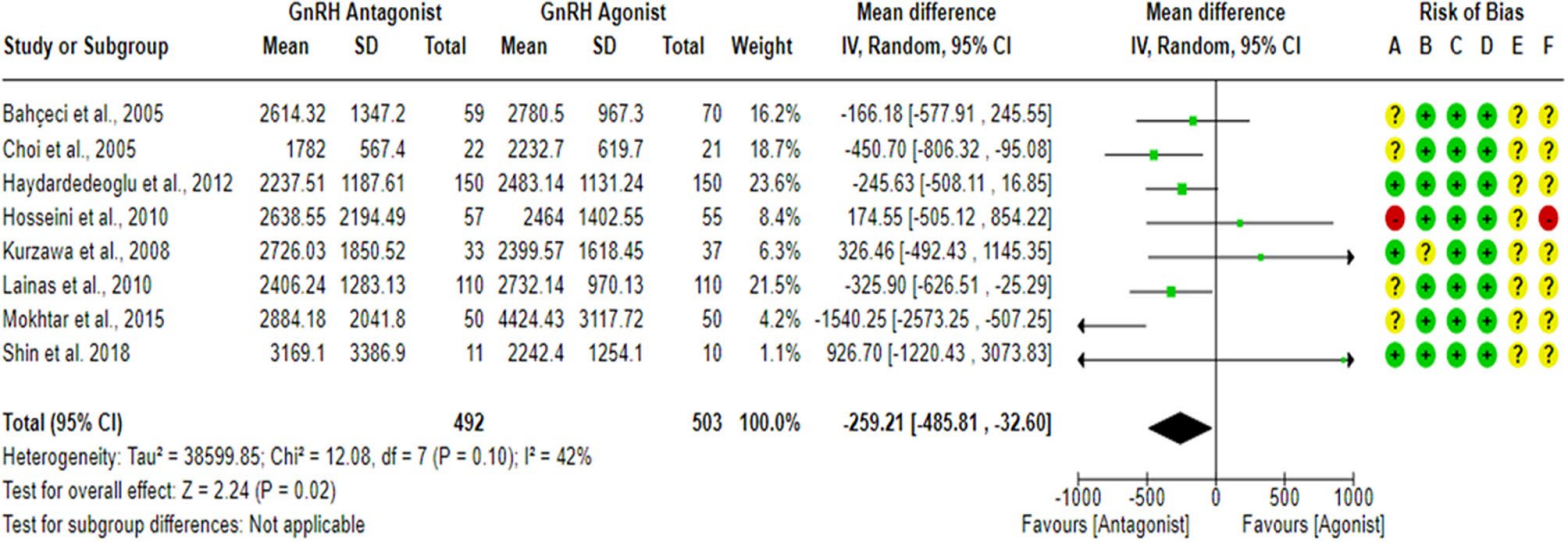
Forest Plot: E2 Levels on hCG Day. (**A**) Bias arising from the randomization process; (**B**) Bias due to deviations from intended interventions; (**C**) Bias due to missing outcome data;(**D**) Bias in measurement of the outcome; (**E**) Bias in selection of the reported result and (**F**) overall bias.

Eight RCTs^18,23,27,43,59–62^ compared E2 levels on hCG day between the GnRH antagonist and GnRH agonist protocols in 995 PCOS women. All included studies were at “low risk” of bias due to deviations from intended interventions except for one study^61^, which held “some concern” since a per-protocol analysis was conducted. However, the number of excluded participants was unlikely to have a substantial impact on the results. All included studies were at “low risk” of bias due to missing outcome data and bias in outcome measurement, taking into account the number of missing outcome data, and the causes of missingness. Moreover, it is an objective outcome, and its assessment would not be influenced by knowledge of the intervention received. Pooling the results of these RCTs showed a significant lower E2 level on hCG day in the GnRH antagonist protocols compared with the Long GnRH agonist protocol (WMD = − 259.21, 95% CI: [− 485.81 to − 32.60] pg/ml; *P* = 0.02; I^2^ = 42%; χ^2^-*P* = 0.10; moderate-quality evidence; Fig. 13). Based on the results of the sensitivity analysis, the results were robust against the exclusion of the high risk of bias study^60^ (WMD = − 298.98, 95% CI: [− 530.64 to − 67.32] pg/ml; *P* = 0.01; I^2^ = 42%; χ^2^-*P* = 0.11) and the removal of estimated outcome data^61,62^ (WMD = − 292.87, 95% CI: [− 591.48 to 5.74] pg/ml; *P* = 0.05; I^2^ = 49%; χ^2^-*P* = 0.08). Using GRADE, we judged the certainty of evidence as moderate; we down-graded it by one level due to concerns over imprecision.

### Endometrial thickness on hCG day

**Figure 14:**
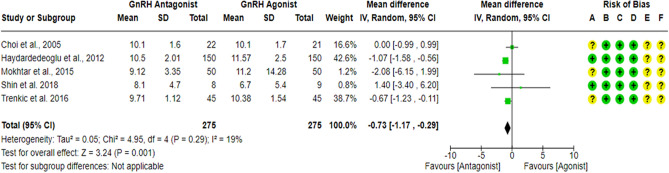
Forest Plot: Endometrial Thickness on hCG Day. (**A**) Bias arising from the randomization process; (**B**) Bias due to deviations from intended interventions; (**C**) Bias due to missing outcome data;(**D**) Bias in measurement of the outcome; (**E**) Bias in selection of the reported result and (**F**) overall bias.

Five RCTs^18,23,27,43,63^ compared Endometrial thickness on hCG day between the GnRH antagonist and GnRH agonist protocols in 550 PCOS women. All included studies were at “low risk” of bias due to missing outcome data, bias due to deviations from intended interventions and bias in outcome measurement, considering the type of analysis, the number of missing outcome data, and the causes of missingness. Moreover, it is an objective outcome, and its assessment would not be influenced by knowledge of the intervention received. Pooling the results of these RCTs showed a significant lower Endometrial thickness on hCG day in the GnRH antagonist protocols compared to the Long GnRH agonist protocol (WMD = -0.73, 95% CI: [-1.17 to -0.29] mm; P = 0.001; I^2^ = 19%; χ^2^-P = 0.29; moderate-quality evidence; Fig. 14). Using GRADE, we judged the certainty of evidence as moderate; we down-graded it by one level due to concerns over imprecision.

### Number of retrieved oocytes

**Figure 15:**
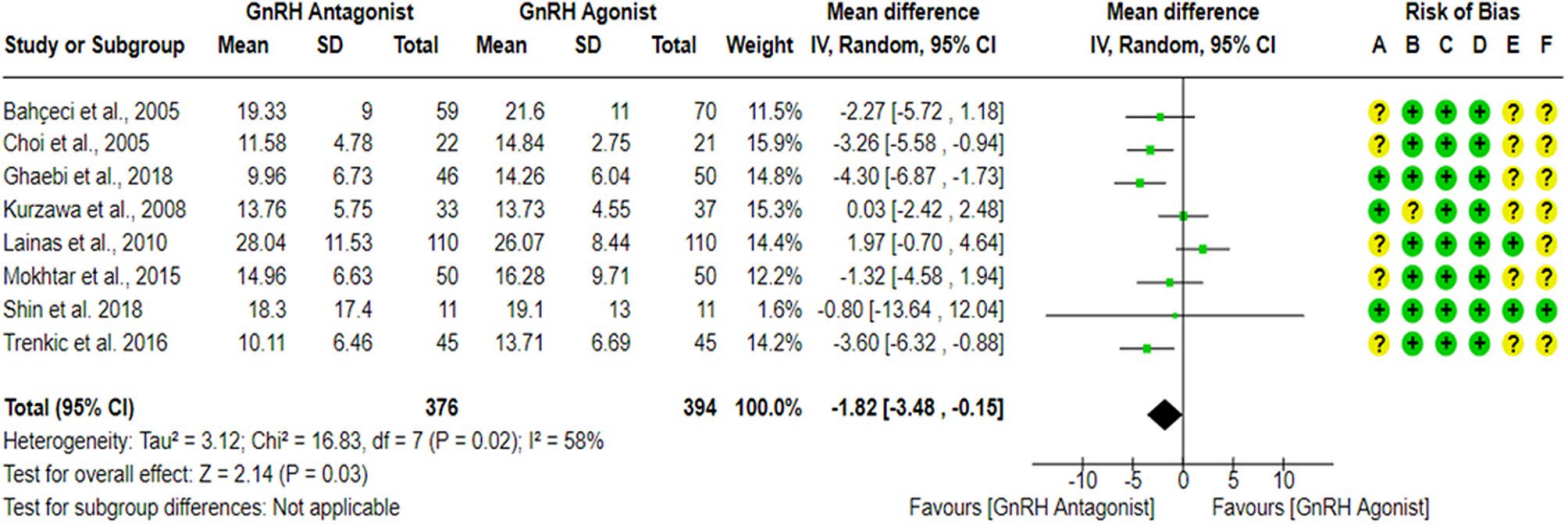
Forest Plot: Number of Retrieved Oocytes. (**A**) Bias arising from the randomization process; (**B**) Bias due to deviations from intended interventions; (**C**) Bias due to missing outcome data;(**D**) Bias in measurement of the outcome; (**E**) Bias in selection of the reported result and (**F**) overall bias.

Nine RCTs^17,23,27,43,59–63^ compared the number of retrieved oocytes between the GnRH antagonist and GnRH agonist protocols in 882 PCOS women. All included studies were at “low risk” of bias due to deviations from intended interventions except for one study^61^, which held “some concern” since a per-protocol analysis was conducted. Yet, the number of excluded participants was unlikely to have a substantial impact on the results. All included studies were at “low risk” of bias due to missing outcome data and bias in outcome measurement, considering the number of missing outcome data, and the causes of missingness. Moreover, it is an objective outcome, and its assessment would not be influenced by knowledge of the intervention received. Only two studies^27,62^ had a prospective registry record, and the outcome was pre-specified in it, so they were at “low risk” of bias in selection of the reported result. Pooling the results of these RCTs did not show any differences in the number of retrieved oocytes between the GnRH antagonist protocols and the Long GnRH agonist protocol (WMD = − 1.13, 95% CI: [− 3.11 to 0.84] oocyte; *P* = 0.26; I^2^ = 75%; χ^2^-*P* < 0.0001). Based on the results of the sensitivity analysis, the results were robust against the removal of estimated outcome data^43,61,62^ (WMD = − 1.55, 95% CI: [− 4.42 to 1.31] oocyte; *P* = 0.29; I^2^ = 78%; χ^2^-*P* = 0.0004). Nevertheless, exclusion of high risk of bias studies^60^ changed the effect, as the results showed a significantly lower number of retrieved oocytes in the GnRH antagonist protocols compared to the Long GnRH agonist protocol (WMD = − 1.82, 95% CI: [− 3.48 to − 0.15] oocytes; *P* = 0.03; I^2^ = 58%; χ^2^-*P* = 0.02; low-quality evidence; Fig. 15), so we excluded it from the meta-analysis. The results of subgroup analysis showed a significantly lower number of retrieved oocytes in the GnRH antagonist protocol compared with the Long GnRH agonist protocol after using Flexible GnRH antagonist protocol (7 RCTs; 748 participants; WMD = − 1.83, 95% CI: [− 3.56 to − 0.10] oocytes; *P* = 0.04; I^2^ = 64%; χ^2^-*P* = 0.01), but not Fixed GnRH antagonist protocol (1 RCTs; 22 participants; WMD = -0.80, 95% CI: [− 13.64 to 12.04] oocytes; *P* = 0.90), but the result of the subgroup analysis was not significant (I^2^ = 0%, χ^2^-*P* = 0.88). Using GRADE, we judged the certainty of evidence as low; we down-graded it by two levels, one level due to concerns over imprecision and one due to concerns over heterogeneity.

**Figure 16:**
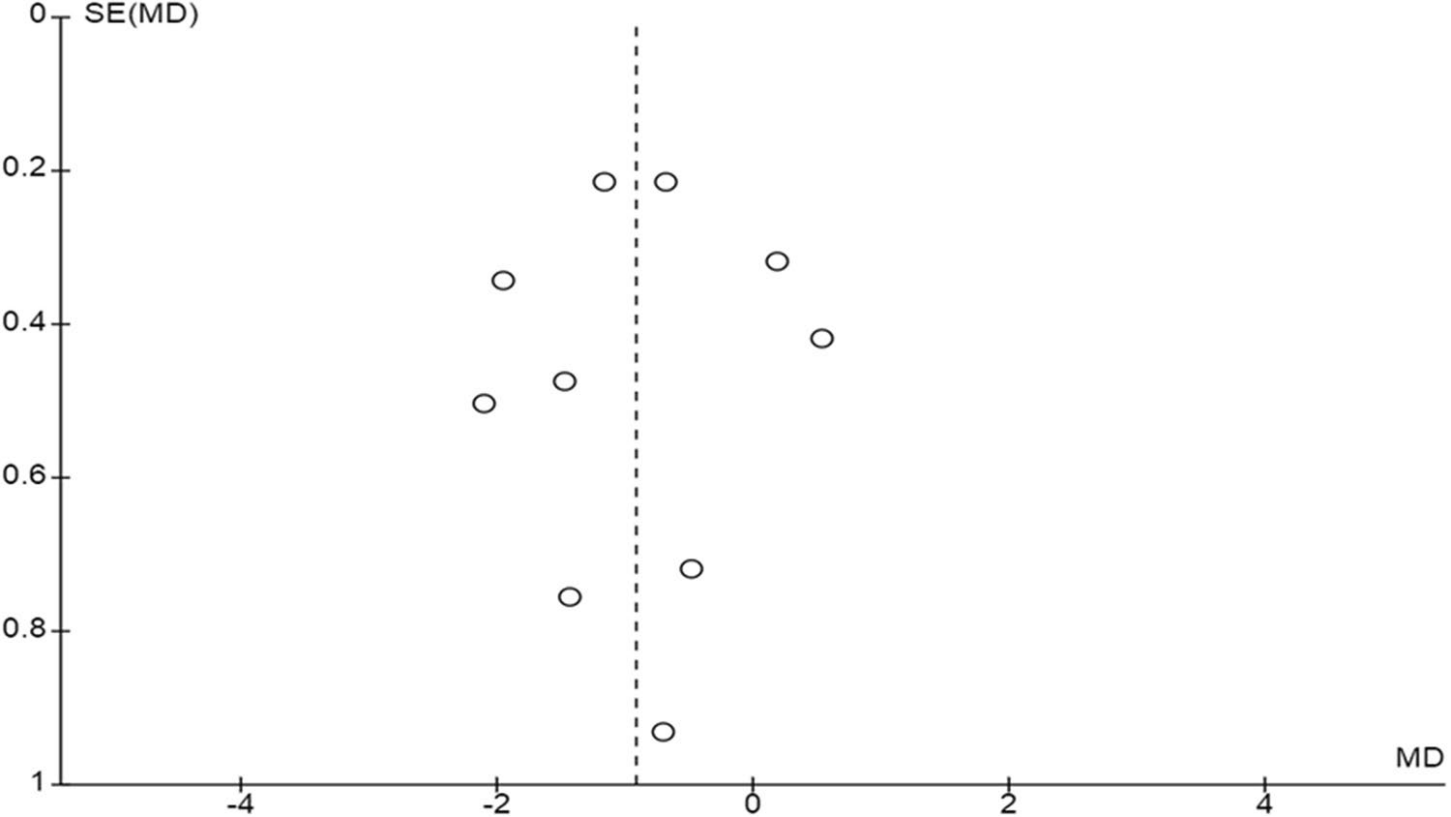
Funnel Plot: Stimulation duration.

Based on the number of included studies in each comparison, we were able to produce only a funnel plot of the stimulation duration outcome, which revealed no publication bias (Fig. 16). The results of our review are summarized in a summary finding table, see Table 2**.**

**Table 2:** Summary of finding table.

Outcome	Anticipated absolute effects (95% CI)		Relative effect (95% CI)	№ of participants (studies)	Certainty
	Risk with Long GnRH agonist	Risk with Conventional GnRH antagonist			
Live birth rate	486 per 1,000	**379 per 1,000** (224 to 642)	**RR 0.78** (0.46 to 1.32)	74 (1 RCT)	⨁⨁◯◯ LOW ^a^
Ongoing pregnancy rate	435 per 1,000	**400 per 1,000** (339 to 470)	**RR 0.92** (0.78 to 1.08)	785 (5 RCTs)	⨁⨁⨁⨁ HIGH
OHSS rate (All grades)	173 per 1,000	**101 per 1,000** (76 to 134)	**RR 0.58** (0.44 to 0.77)	994 (9 RCTs)	⨁⨁◯◯ LOW ^b,c^
Mild OHSS rate	204 per 1,000	**165 per 1,000** (98 to 279)	**RR 0.81** (0.48 to 1.37)	229 (3 RCTs)	⨁◯◯◯ VERY LOW ^a,b^
Moderate-Severe OHSS rate	196 per 1,000	**127 per 1,000** (102 to 161)	**RR 0.65** (0.52 to 0.82)	1114 (9 RCTs)	⨁◯◯◯ VERY LOW ^b,d^
Clinical pregnancy rate	415 per 1,000	**399 per 1,000** (320 to 494)	**RR 0.96** (0.77 to 1.19)	840 (8 RCTs)	⨁⨁⨁⨁ HIGH
Multiple pregnancy rate	99 per 1,000	**86 per 1,000** (58 to 125)	**RR 0.87** (0.59 to 1.27)	994 (9 RCTs)	⨁◯◯◯ VERY LOW ^b,d^
Miscarriage rate	86 per 1,000	**80 per 1,000** (53 to 123)	**RR 0.93** (0.61 to 1.43)	997 (7 RCTs)	⨁◯◯◯ VERY LOW ^a,b^
Cycle cancellation rate	54 per 1,000	**62 per 1,000** (37 to 103)	**RR 1.15** (0.69 to 1.91)	1002 (8 RCTs)	⨁◯◯◯ VERY LOW ^a,b^
Cycle cancellation rate due to high risk of OHSS	32 per 1,000	**19 per 1,000** (8 to 43)	**RR 0.59** (0.26 to 1.34)	869 (6 RCTs)	⨁◯◯◯ VERY LOW ^a,b^
Cycle cancellation rate due to poor ovarian response	7 per 1,000	**32 per 1,000** (10 to 99)	**RR 4.63** (1.49 to 14.41)	869 (6 RCTs)	⨁◯◯◯ VERY LOW ^b,e^
Duration of stimulation	The mean duration of stimulation ranged from **9.07–13.92** Days	MD **0.91 Days. Lower** (1.45 lower to 0.37 lower)	–	1182 (10 RCTs)	⨁◯◯◯ VERY LOW ^f,g^
Gonadotropin Dose	The mean gonadotropin Dose ranged from **2675.5–1370.16** IUs	MD **221.36 IUs. Lowe**r (332.28 lower to 110.45 lower)	–	845 (7 RCTs)	⨁⨁⨁⨁ HIGH
E2 levels hCG day	The mean E2 levels day of hCG ranged from **2232.7–4424.43** pg/ml	MD **259.21 pg/ml. lower** (485.81 lower to 32.6 lower)	–	995 (8 RCTs)	⨁⨁⨁◯ MODERATE ^g^
Endometrial thickness on hCG day	The mean endometrial thickness on day of hCG ranged from **6.7–11.57** mm	MD **0.73 mm. lower** (1.17 lower to 0.29 lower)	–	550 (5 RCTs)	⨁⨁⨁◯ MODERATE ^g^
Number of retrieved oocytes	The mean no. of retrieved oocytes ranged from **13.71–26.07** oocytes	MD **1.82 oocytes. Lower** (3.48 lower to 0.15 lower)	–	770 (8 RCTs)	⨁⨁◯◯ LOW ^g,h^

**E2**: Estradiol, **GnRH**: Gonadotropin-releasing hormone, **hCG**: Human chorionic gonadotropin, **OHSS**: ovarian hyperstimulation syndrome.

^**a**^Evidence downgraded by two levels for very serious imprecision – 95% CI includes both appreciable benefit and harm or no effect and very low number of events (total number of events < 300).

^**b**^Evidence downgraded by one level for serious risk of bias – the majority of the RCTs have some concern or high risk of bias.

^**c**^Evidence downgraded by one level for serious imprecision – low number of events (total number of events < 300).

^**d**^Evidence downgraded by two levels for very serious imprecision – 95% CI includes both appreciable effect and little or no effect and very low number of events (total number of events < 300).

^**e**^Evidence downgraded by two levels for very serious imprecision—low number of events (total number of events < 300) and the effect estimate has a wide confidence interval.

^**f**^Evidence downgraded by two levels for very serious inconsistency (unexplained heterogeneity).

^**g**^Evidence downgraded by one level for serious imprecision – 95% CI includes both appreciable effect and little or no effect.

^**h**^Evidence downgraded by one level for serious inconsistency (unexplained heterogeneity).

## Discussion

The results of our review suggest that using Conventional GnRH antagonist protocol in PCOS women is associated with lower gonadotropins consumption (high-quality evidence), shorter stimulation duration (very low-quality evidence), thinner endometrial thickness on hCG day (moderate-quality evidence), lower E2 levels on hCG day (moderate-quality evidence), lower number of retrieved oocytes (low-quality evidence), and fewer OHSS incidence (low-quality evidence) without compromising clinical pregnancy rate (high-quality evidence), ongoing pregnancy rate (high-quality evidence) or live birth rate (low-quality evidence). Moreover, similar MPR (very low-quality evidence) and MR (very low-quality evidence) have been noticed in the GnRH antagonist protocols and the Long GnRH agonist protocol. Similarly, the overall risk of cycle cancellation is comparable between the two groups (very low-quality evidence). Nevertheless, more cycles have been cancelled due to poor ovarian response in the GnRH antagonist protocols (very low-quality evidence), while similar rates of cancellation due to risk of OHSS have been observed in both groups (very low-quality evidence).

Lack of gonadotropins suppression during the early follicular phase of Conventional GnRH antagonist protocols may lay behind the shorter stimulation duration, the lower gonadotropins consumption, and the lower number of retrieved oocytes noted in the GnRH antagonist group compared to the Long GnRH agonist one since higher LH levels may improve FSH sensibility^61^, while higher FSH leads to an uncoordinated and a heterogeneous development of FSH‐sensitive follicles^20,21^, which may, as a result, reduce the number of retrieved oocytes. However, it is worth mentioning that all included women were pre-treated with oral contraceptive pills (OCPs). Although OCPs effects on IVF outcomes are still unclear and controversial, they may prevent the early rise of endogenous gonadotropins during the Conventional GnRH antagonist protocol and improve the homogeneity of the follicular development^64^. However, Although it was suggested that a 5-days OCPs free interval is enough for gonadotropins recovery^65^, Kolibianakis et al.^66^ observed significantly lower levels of LH, E2 and progesterone after five days of OCPs cessation compared with the levels observed in the same patients at the time of OCPs initiation, and compared with the levels seen in patients underwent GnRH antagonist COS without OCPs on Day2 cycle. Further, the LH levels remained lower in the OCPs group till the day of hCG triggering^66^. However, differences in the ovarian microenvironment can also be suspected. Arising evidence highlight the possibility of GnRH analogues affecting ovarian cells directly^67,68^. Several studies have reported different steroidogenesis pattern in GnRH antagonist and GnRH agonist protocols^69–72^. However, It is unlikely that the differences in steroidogenesis arise from differences in the steroidogenic cell numbers, as similar levels of granulosa cells apoptosis were observed in the GnRH antagonist protocols and the GnRH agonist ones^69,73,74^. Previously, Khalaf et al.^69^ noted that granulosa lutein cells obtained from GnRH antagonist-treated women showed significantly lower aromatase activity, lower aromatase expression, and higher expression of FSH receptors compared to those obtained from GnRH agonist-treated women. Similarly, Winkler et al.^70^ noted that GnRH antagonist led to a dose-dependent reduction in granulosa cell aromatase in a granulosa cell culture model. On the contrary, the GnRH agonist stimulated aromatase in a dose-dependent manner. These effects combined with the possible ability of GnRH antagonists to reduce the number of retrieved oocytes may be responsible for their protective effect in reducing OHSS incidences during COS. In addition, Vrtačnik-Bokal et al. study^49^ reported lower estradiol and vascular endothelial growth factors (VEGF) levels in the follicular fluids (FF) of PCOS women treated with the GnRH antagonist protocol compared to those treated with the Long GnRH agonist protocol. On the contrary, Ferrari et al. study^75^ showed higher FF VEGF levels in the GnRH antagonist group compared to the Long GnRH agonist one. On the other side, Malhotra et al.^76^ observed higher FF VEGF levels in normo-responder women treated with the GnRH antagonist protocol compared to the Long GnRH agonist protocol. Yet, the ratio of the soluble form of VEGF receptor-1 (sVEGFR-1) to VEGF did not differ between the two protocols significantly. VEGF, also called vascular permeability factor, is the founding member of the VEGFs family, which is known for its role in regulating angiogenesis and vasculogenesis^77^. The soluble form of VEGF receptor-1 (sVEGFR-1) acts as an anti-angiogenic factor by sequestering VEGF and decreasing its free form availability^78^. Arising evidence supports VEGF pivotal role in regulation follicle angiogenesis^79^, ovulation^80,81^, placentation and implantation^82,83^. Moreover, VEGF plays an important role in the pathophysiology of OHSS^13,84^. PCOS ovaries exhibit higher vascularization and lower impedance to flow in ovarian stromal vessels compared to control^85–87^, which may be arisen from the differences in the levels of ovarian angiogenesis regulation factors as PCOS women showed higher VEGF levels and lower sVEGFR-1 levels compared to control both in serum and follicular fluid samples^88,89^. Based on the abovementioned results, GnRH antagonist effects on reducing VEGF levels during COS may be more noticeable in the PCOS subjects due to the ovarian angiogenesis abnormality seen in this population. However, with such limited data, it is risky to conclude that similar effects unlikely to be seen in non-PCOS subjects. Thus, Further research is needed to confirm this hypothesis and provide a better understanding of the mechanism that underlies GnRH antagonists’ protective effects in reducing OHSS incidences during COS.

The effect of GnRH antagonists on endometrial receptivity is still questionable. Several studies suggested a detrimental impact on endometrial receptivity^90–93^. Some reports noticed a significant reduction in the endometrial expression of HOXA-10 during GnRH antagonist cycles compared with GnRH agonist cycles or natural cycles^90,91^. HOXA10 is a transcription factor that belongs to the homeobox gene family. It is highly expressed in endometrial stromal cells and plays essential roles in embryo implantation and endometrium proliferation, differentiation, and receptivity^94^. Infertility due to implantation failure was noted in female mice with HOXA-10 mutation. However, these mice gave viable embryos that could implant and develop normally in a wild-type surrogate^95^. Moreover, a recent study showed an upregulation of endometrial Allograft inflammatory factor-1 (AIF-1) expression, a cytokine associated with inflammation and allograft rejection, in the GnRH antagonist group compared with the GnRH agonist one, which might be unfavorable for embryo implantation as increased AIF-1 might inhibit adhesion during implantation via raised Tumor necrosis factor-α (TNF-α)^92^. Furthermore, although treating mice with both GnRH agonist and GnRH antagonist was associated with a reduction in the endometrial expression of the endometrial receptivity markers; leukemia-inhibitory factor (LIF) and integrin β3, GnRH agonist-treated mice showed a higher implantation rate and a higher endometrial expression of LIF and integrin β3 subunit^93^. On the other hand, several clinical studies revealed that the endometrial development during GnRH antagonist cycles mimics the natural endometrium more closely than GnRH agonist cycles^96,97^. However, it still unknown whether the GnRH antagonists may have similar effects on PCOS subjects who suffered from abnormal endometrial receptivity^98,99^. Our review showed a significant reduction in endometrial thickness in the GnRH antagonist group compared with the GnRH agonist one. In practice, CPR and LBR decrease for each millimeter of endometrial thickness declines below 8 mm in fresh IVF cycles^100^. Yet, endometrial thickness means in the GnRH antagonist group range from 8.1 to 10.5 mm VS 6.7 to 11.57 mm in the GnRH agonist group, with mean differences -0.73 mm and 95% CI [-1.17 to -0.29] mm. Thus, it is unlikely that the effect on endometrial thickness will lead to clinically meaningful adverse effects on CPR, OPR, MR or LBR in general cases.

The results of previous reviews showed no differences in CPR^28–32^, OPR^28,29^, LBR^29^, MR^30,31^, and CCR due to high risk of OHSS^31^ between PCOS subjects treated with GnRH antagonist protocols and the Long GnRH agonist protocol, which comes along with the results of our review. In agreement with our results, Lambalk et al.^29^ and Xiao et al.^30^ studies showed that using GnRH antagonist in PCOS subjects is associated with lower OHSS incidences. Moreover, Pundir et al.^31^ also reported lower incidences of Severe-Moderate OHSS but not Mild OHSS in the GnRH antagonist protocols, which also comes along with our results. Furthermore, we noted lower gonadotropins consumption and shorter stimulation duration in GnRH antagonist protocols compared with the Long GnRH agonist protocol, which follows previous review results^28,31^. However, Lin et al.^28^ and Griesinger et al.^32^ did not note significant differences in OHSS rate between GnRH antagonist protocols and the Long GnRH agonist protocol. Moreover, Griesinger et al.^32^ did not observe any significant differences in the gonadotropins consumption between different GnRH analogues protocols. This may be due to the limited number of studies were included in the meta-analyses that investigated these effects in those reviews. On the contrary with our results, previous reviews did not show any significant differences between GnRH antagonist protocols and GnRH agonist protocols on E2 levels on hCG day^30,31^ or the number of retrieved oocytes^29–32^ except for Lin et al.^28^ review, which reported lower retrieved oocytes number in the GnRH antagonist protocols, which may arise from the differences in studies inclusion and exclusion criteria since all previous reviews included both Early and Conventional GnRH antagonist protocols, while ours only included Conventional protocols.

### Strengths, limitations and future research

The main strength of the present study is being the first systematic review and meta-analysis to investigate the effects of Conventional GnRH antagonist protocols and the Long GnRH agonist protocol on IVF/ICSI outcomes in women with PCOS. In addition, we examined the effects of GnRH analogues on twelve IVF/ICSI outcomes and assessed the quality of the obtained evidence using the GRADE approach, which gives a better understanding of the overall effects and aids the health care providers to improve the plan therapy offered to patients. However, there were also some limitations. Although we conducted a comprehensive search of relevant databases besides the reference lists hand-search with no language restrictions, we may have missed trials that would have been eligible for inclusion. Furthermore, despite the results tables of the non-English published studies could directly be obtained and interpreted, assessing the methodological quality was a bit challenging. Another limitation is the number of included studies since it was relatively small, and the obtained evidence was downgraded for imprecision for several outcomes. For the same reason, we were incapable of assessing the risk of publication bias using funnel plots except for one outcome (stimulation duration). Above that, the diagnosis criteria of OHSS and CCR were poorly defined among the included trials. Also, despite our attempts to obtain the missing outcome data from the study authors, some data were still missing, and they possibly caused some bias. In addition, all included studies focused on fresh cycles. Thus, the reproducibility of obtained results in frozen cycles is still unknown. Therefore, more well-designed RCTs following the CONSORT statement are required to be more confidant in the results obtained on PCOS women during fresh cycles and to further compare the effects of the Conventional GnRH antagonist protocols and the GnRH agonist protocols on IVF/ICSI outcomes during frozen ones.

## Conclusions

Conventional GnRH antagonist protocols represent a safer and more cost-effective choice for PCOS women undergoing IVF/ICSI cycles than the standard Long GnRH agonist protocol without compromising the IVF/ICSI clinical outcomes.

## Supplementary Information

Supplementary Information.

## Data Availability

All the data supporting the findings of this study are available within the article and its supplementary material.
