# Supplementary material for: Conventional GnRH antagonist protocols versus long GnRH agonist protocol in IVF/ICSI cycles of polycystic ovary syndrome women: a systematic review and meta-analysis
Source: Sci Rep. 2022 Mar 15;12:4456. doi: 10.1038/s41598-022-08400-z (PMC8924277; doi:10.1038/s41598-022-08400-z)
Supplement: Supplementary file 1 — Supplementary Information. [file 41598_2022_8400_MOESM1_ESM.pdf]

Conventional GnRH antagonist protocols Versus long GnRH agonist protocol in IVF/ICSI cycles of polycystic ovary syndrome women: a systematic review and meta-analysis.

Sally Kadoura* [1], Marwan Alhalabi [2,3], Abdul Hakim Nattouf [1].

[1] Department of Pharmaceutics and Pharmaceutical Technology, Faculty of Pharmacy, Damascus University, Damascus, Syrian Arab Republic.

[2] Department of Embryology and Reproductive Medicine, Faculty of Medicine, Damascus University, Damascus, Syrian Arab Republic.

[3] Assisted Reproduction Unit, Orient Hospital, Damascus, Syrian Arab Republic.

Supplementary Table S1: PRISMA Abstract checklist 2020 [1]:

Section and Topic	Item #	Checklist item	Reported (Yes/No)
**TITLE**			
Title	1	Identify the report as a systematic review.	**Yes.**
**BACKGROUND**			
Objectives	2	Provide an explicit statement of the main objective(s) or question(s) the review addresses.	**Yes.**
**METHODS**			
Eligibility criteria	3	Specify the inclusion and exclusion criteria for the review.	**Yes.**
Information sources	4	Specify the information sources (e.g. databases, registers) used to identify studies and the date when each was last searched.	**Yes.**
Risk of bias	5	Specify the methods used to assess risk of bias in the included studies.	**Yes.**
Synthesis of results	6	Specify the methods used to present and synthesise results.	**Yes.**
**RESULTS**			
Included studies	7	Give the total number of included studies and participants and summarise relevant characteristics of studies.	**Yes.**
Synthesis of results	8	Present results for main outcomes, preferably indicating the number of included studies and participants for each. If meta-analysis was done, report the summary estimate and confidence/credible interval. If comparing groups, indicate the direction of the effect (i.e. which group is favoured).	**Yes.**
**DISCUSSION**			
Limitations of evidence	9	Provide a brief summary of the limitations of the evidence included in the review (e.g. study risk of bias, inconsistency and imprecision).	**Yes.**
Interpretation	10	Provide a general interpretation of the results and important implications.	**Yes.**
**OTHER**			
Funding	11	Specify the primary source of funding for the review.	**Yes.**
Registration	12	Provide the register name and registration number.	**Yes.**

Supplementary Table S2: PRISMA checklist 2020 [1]:

Section and Topic	Item #	Checklist item	Location where item is reported (Paragraph)
**TITLE**			
Title	1	Identify the report as a systematic review.	• **Title.**
**ABSTRACT**			
Abstract	2	See the PRISMA 2020 for Abstracts checklist.	• **Abstract.**
**INTRODUCTION**			
Rationale	3	Describe the rationale for the review in the context of existing knowledge.	• **Introduction.**
Objectives	4	Provide an explicit statement of the objective(s) or question(s) the review addresses.	• **Introduction.**
**METHODS**			
Eligibility criteria	5	Specify the inclusion and exclusion criteria for the review and how studies were grouped for the syntheses.	• **Eligibility criteria.** • **Exclusion criteria.**
Information sources	6	Specify all databases, registers, websites, organisations, reference lists and other sources searched or consulted to identify studies. Specify the date when each source was last searched or consulted.	• **Information sources and Search strategies.**
Search strategy	7	Present the full search strategies for all databases, registers and websites, including any filters and limits used.	• **Supplementary Table S3.**
Selection process	8	Specify the methods used to decide whether a study met the inclusion criteria of the review, including how many reviewers screened each record and each report retrieved, whether they worked independently, and if applicable, details of automation tools used in the process.	• **Selection Process.**
Data collection process	9	Specify the methods used to collect data from reports, including how many reviewers collected data from each report, whether they worked independently, any processes for obtaining or confirming data from study investigators, and if applicable, details of automation tools used in the process.	• **Data collection process and Data items.**
Data items	10a	List and define all outcomes for which data were sought. Specify whether all results that were compatible with each outcome domain in each study were sought (e.g. for all measures, time points, analyses), and if not, the methods used to decide which results to collect.	• **Data collection process and Data items.**
	10b	List and define all other variables for which data were sought (e.g. participant and intervention characteristics, funding sources). Describe any assumptions made about any missing or unclear information.	• **Data collection process and Data items.**
Study risk of bias assessment	11	Specify the methods used to assess risk of bias in the included studies, including details of the tool(s) used, how many reviewers assessed each study and whether they worked independently, and if applicable, details of automation tools used in the process.	• **Assessment of risk of bias in included studies.**
Effect measures	12	Specify for each outcome the effect measure(s) (e.g. risk ratio, mean difference) used in the synthesis or presentation of results.	• **Summary measures.**
Synthesis methods	13a	Describe the processes used to decide which studies were eligible for each synthesis (e.g. tabulating the study intervention	• **Data Synthesis.**

		characteristics and comparing against the planned groups for each synthesis (item #5)).	
	13b	Describe any methods required to prepare the data for presentation or synthesis, such as handling of missing summary statistics, or data conversions.	• **Dealing with missing data.**
	13c	Describe any methods used to tabulate or visually display results of individual studies and syntheses.	• **Data Synthesis.**
	13d	Describe any methods used to synthesize results and provide a rationale for the choice(s). If meta-analysis was performed, describe the model(s), method(s) to identify the presence and extent of statistical heterogeneity, and software package(s) used.	• **Data Synthesis.**
	13e	Describe any methods used to explore possible causes of heterogeneity among study results (e.g. subgroup analysis, meta-regression).	• **Data Synthesis.**
	13f	Describe any sensitivity analyses conducted to assess robustness of the synthesized results.	• **Data Synthesis.**
Reporting bias assessment	14	Describe any methods used to assess risk of bias due to missing results in a synthesis (arising from reporting biases).	• **Publication bias assessment.**
Certainty assessment	15	Describe any methods used to assess certainty (or confidence) in the body of evidence for an outcome.	• **Assessment of Certainty of evidence.**
**RESULTS**			
Study selection	16a	Describe the results of the search and selection process, from the number of records identified in the search to the number of studies included in the review, ideally using a flow diagram.	• **Study selection.**<br>• **Figure 1. Flow diagram selection process.**
	16b	Cite studies that might appear to meet the inclusion criteria, but which were excluded, and explain why they were excluded.	• **Study selection.**<br>• **Table 1. Excluded studies.**
Study characteristics	17	Cite each included study and present its characteristics.	• **Study characteristics.**<br>• **Supplementary Table S4.**
Risk of bias in studies	18	Present assessments of risk of bias for each included study.	• **Risk of bias of the included studies (for bias arising from the randomization process).**<br>• **Other types of risk of bias were investigated at outcome-level and discussed in each outcome's paragraph.**
Results of individual studies	19	For all outcomes, present, for each study: (a) summary statistics for each group (where appropriate) and (b) an effect estimate and its precision (e.g. confidence/credible interval), ideally using structured tables or plots.	• **A forest plot was formed to summarize the results of included studies for each outcome and put in the "Results" section.**
Results of syntheses	20a	For each synthesis, briefly summarise the characteristics and risk of bias among contributing studies.	• **This was reported at an outcome-level. Each outcome was discussed separately in the "Results" section in an individual paragraph, and a summary of the risk of**

			**bias assessment was also included in the forest plots.**
	20b	Present results of all statistical syntheses conducted. If meta-analysis was done, present for each the summary estimate and its precision (e.g. confidence/credible interval) and measures of statistical heterogeneity. If comparing groups, describe the direction of the effect.	**• This was reported at an outcome-level. Each outcome was discussed separately in the "Results" section in an individual paragraph, and the results were summarized in the forest plots.**
	20c	Present results of all investigations of possible causes of heterogeneity among study results.	**• This was reported at an outcome-level (when necessary). Each outcome was discussed separately in the "Results" section in an individual paragraph.**
	20d	Present results of all sensitivity analyses conducted to assess the robustness of the synthesized results.	**• This was reported at an outcome-level (when necessary). Each outcome was discussed separately in the "Results" section in an individual paragraph.**
Reporting biases	21	Present assessments of risk of bias due to missing results (arising from reporting biases) for each synthesis assessed.	**• We were incapable of assessing the risk of publication bias using funnel plots for any outcomes due to the limited number of included studies.**
Certainty of evidence	22	Present assessments of certainty (or confidence) in the body of evidence for each outcome assessed.	**• This was reported at an outcome-level. Each outcome was discussed separately in the "Results" section in an individual paragraph.** **• Table 2. Summary of finding table.**
**DISCUSSION**			
Discussion	23a	Provide a general interpretation of the results in the context of other evidence.	**• Discussion.**
	23b	Discuss any limitations of the evidence included in the review.	**• Limitations.**
	23c	Discuss any limitations of the review processes used.	**• Limitations.**
	23d	Discuss implications of the results for practice, policy, and future research.	**• Conclusions.**
**OTHER INFORMATION**			
Registration and protocol	24a	Provide registration information for the review, including register name and registration number, or state that the review was not registered.	**• Protocol and registration, PROSPERO**

			**(CRD42021242476).**
	24b	Indicate where the review protocol can be accessed, or state that a protocol was not prepared.	• **Protocol and registration, PROSPERO (CRD42021242476).**
	24c	Describe and explain any amendments to information provided at registration or in the protocol.	• **Protocol and registration, PROSPERO (CRD42021242476).**
Support	25	Describe sources of financial or non-financial support for the review, and the role of the funders or sponsors in the review.	• **Funding.**
Competing interests	26	Declare any competing interests of review authors.	• **Conflicts of interest.**
Availability of data, code and other materials	27	Report which of the following are publicly available and where they can be found: template data collection forms; data extracted from included studies; data used for all analyses; analytic code; any other materials used in the review.	• **Data Availability.**

Supplementary Table S3: Search Strategy:

Cochrane Central Register of Controlled Trials (CENTRAL)

#1	MeSH descriptor: [Polycystic Ovary Syndrome] explode all trees	1520
#2	(polycystic ovar* syndrome):ti,ab,kw OR (polycystic ovar* disease):ti,ab,kw OR (PCOD):ti,ab,kw OR (PCOS):ti,ab,kw OR (PCO):ti,ab,kw	4620
#3	(Stein Leventhal syndrome):ti,ab,kw OR (sclerocystic ovar* syndrome):ti,ab,kw OR (Sclerocystic Ovar* Degeneration):ti,ab,kw	42
#4	#1 or #2 or #3	4621
#5	(assisted reproducti* techn*):ti,ab,kw OR (reproducti* medic*):ti,ab,kw OR (fertili?ation in vitro):ti,ab,kw OR (IVF):ti,ab,kw OR (test-tube fertili?ation):ti,ab,kw	9880
#6	(test-tube baby):ti,ab,kw OR (intracytoplasmic sperm injection):ti,ab,kw OR (ICSI):ti,ab,kw OR (artificial insemination):ti,ab,kw OR (ovulat* stimulat*):ti,ab,kw	5493
#7	(ovulat* induc*):ti,ab,kw AND (ovulat* hyperstimulat*):ti,ab,kw AND (ovar* stimulat*):ti,ab,kw AND (ovar* induc*):ti,ab,kw AND (ovar* hyperstimulat*):ti,ab,kw	528
#8	(control* ovar* stimulat*):ti,ab,kw OR (control* ovar* hyperstimulat*):ti,ab,kw OR (superovulat*):ti,ab,kw	3927
#9	(COS):ti,ab,kw OR (COH):ti,ab,kw OR (oocyte* retrieval*):ti,ab,kw OR (embryo* transfer*):ti,ab,kw OR (IVF-ET):ti,ab,kw	6053
#10	MeSH descriptor: [Reproductive Techniques, Assisted] explode all trees	3273
#11	MeSH descriptor: [Reproductive Medicine] explode all trees	171
#12	#5 or #6 or #7 or #8 or #9 or #10 or #11	14327
#13	MeSH descriptor: [Gonadotropin-Releasing Hormone] explode all trees	2647
#14	(gonadotrop?in-releasing hormone):ti,ab,kw OR (gonadotrop?in releasing hormone):ti,ab,kw OR (Gonadoliberin):ti,ab,kw OR (Luliberin):ti,ab,kw OR (Gonadorelin):ti,ab,kw	3206
#15	(LH-FSH Releasing Hormone):ti,ab,kw OR (LH Releasing Hormone):ti,ab,kw OR (FSH Releasing Hormone):ti,ab,kw OR (LHRH):ti,ab,kw	1945
#16	(GNRH):ti,ab,kw OR (GN-RH):ti,ab,kw OR (LH-RH):ti,ab,kw OR (LHFSHRH):ti,ab,kw OR (LFRH):ti,ab,kw	3795
#17	(antagonist*):ti,ab,kw OR (inhibitor*):ti,ab,kw	127573
#18	(GnRH-anta):ti,ab,kw OR (GnRHant):ti,ab,kw OR (GnRHanta):ti,ab,kw OR (GnRH ant):ti,ab,kw OR (GnRH anta):ti,ab,kw	59
#19	(cetrorelix):ti,ab,kw OR (cetrolix):ti,ab,kw OR (cetrorelix acetate):ti,ab,kw OR (cetrorelix pamoate):ti,ab,kw OR (cetrotide):ti,ab,kw	389
#20	(abarelix):ti,ab,kw OR (plenaxis):ti,ab,kw OR (relugolix):ti,ab,kw OR (GnRH-ant):ti,ab,kw	49
#21	(ganirelix):ti,ab,kw OR (ganirelix acetate):ti,ab,kw OR (antagon):ti,ab,kw OR (orgalutran):ti,ab,kw	241
#22	#13 or #14 or #15 or #16	6357
#23	#22 and #17	2426
#24	#23 or #18 or #19 or #20 or #21	2592

#25	(agonist*):ti,ab,kw	25691
#26	(GnRH-a):ti,ab,kw OR (GnRHa):ti,ab,kw OR (GnRH a):ti,ab,kw	3217
#27	(buserelin):ti,ab,kw OR (busereline):ti,ab,kw OR (buserelin acetate):ti,ab,kw OR (suprefact):ti,ab,kw OR (profact):ti,ab,kw	537
#28	(receptal):ti,ab,kw OR (tiloryth):ti,ab,kw OR (suprecur):ti,ab,kw OR (bigonist):ti,ab,kw	4
#29	MeSH descriptor: [Buserelin] explode all trees	292
#30	(goserelin):ti,ab,kw OR (gosereline):ti,ab,kw OR (goserelin acetate):ti,ab,kw OR (Zoladex):ti,ab,kw	1168
#31	MeSH descriptor: [Goserelin] explode all trees	575
#32	(nafarelin):ti,ab,kw OR (nafareline):ti,ab,kw OR (nafarelin acetate):ti,ab,kw OR (Synarel):ti,ab,kw	146
#33	MeSH descriptor: [Nafarelin] explode all trees	77
#34	(triptoielin):ti,ab,kw OR (triptoreline):ti,ab,kw OR (triptorelin pamoate):ti,ab,kw OR (triptrolein):ti,ab,kw OR (triptorelyn):ti,ab,kw	502
#35	(Decapeptyl):ti,ab,kw	143
#36	MeSH descriptor: [Triptorelin Pamoate] explode all trees	455
#37	(leuprorelin):ti,ab,kw OR (leuprolin):ti,ab,kw OR (leuprorelin acetate):ti,ab,kw OR (leuprolide):ti,ab,kw OR (leuprolide acetate):ti,ab,kw	1264
#38	(Enantone):ti,ab,kw OR (Lupron):ti,ab,kw	101
#39	MeSH descriptor: [Leuprolide] explode all trees	694
#40	#22 AND #25	3073
#41	#26 or #27 or #28 or #29 or #30 or #31 or #32 or #33 or #34 or #35 or #36 or #37 or #38 or #39 or #40	6213
#42	#4 and #12 and #24 and #41	136

Pubmed Database

#1	"polycystic ovary syndrome"[MeSH Terms] OR ("polycystic"[All Fields] AND ("ovary"[All Fields] OR "ovarian"[All Fields]) AND ("syndrome"[All Fields] OR "disease"[All Fields])) OR "polycystic ovary syndrome"[All Fields] OR "polycystic ovarian syndrome"[All Fields] OR "polycystic ovary disease"[All Fields] OR "polycystic ovarian disease"[All Fields] OR (("stein"[All Fields] AND "leventhal"[All Fields] AND "syndrome"[All Fields]) OR "stein leventhal syndrome"[All Fields]) OR ("sclerocystic"[All Fields] AND ("ovary"[All Fields] OR "ovarian"[All Fields]) AND ("syndrome"[All Fields] OR "degeneration"[All Fields])) OR "sclerocystic ovary syndrome"[All Fields] OR "sclerocystic ovarian syndrome"[All Fields] OR "sclerocystic ovary degeneration"[All Fields] OR "sclerocystic ovarian degeneration"[All Fields] OR "PCOS"[All Fields] OR "PCOD"[All Fields] OR "PCO"[All Fields]	25,089
#2	"reproductive techniques, assisted"[MeSH Terms] OR ("reproductive"[All Fields] AND "techniques"[All Fields] AND "assisted"[All Fields]) OR "assisted reproductive techniques"[All Fields] OR ("assisted"[All Fields] AND "reproductive"[All Fields] AND "technique"[All Fields]) OR "assisted reproductive technique"[All Fields] OR "reproductive medicine"[MeSH Terms] OR (("reproductive"[All Fields] OR "reproduction"[All Fields]) AND "medicine"[All Fields]) OR "reproductive medicine"[All Fields] OR "reproduction medicine"[All Fields] OR ("fertilization"[All Fields] AND "vitro"[All Fields]) OR "fertilization in vitro"[All Fields] OR ("fertilisation"[All Fields] AND "vitro"[All Fields]) OR "fertilisation in vitro"[All Fields] OR (("test"[All Fields] AND "tube"[All Fields] AND "fertilization"[All Fields]) OR "test tube fertilization"[All Fields]) OR (("test"[All Fields] AND "tube"[All Fields] AND "fertilisation"[All Fields]) OR "test tube fertilisation"[All Fields]) OR "ivf"[All Fields] OR (("test"[All Fields] AND "tube"[All Fields] AND "baby"[All Fields]) OR "test tube	288,059

	baby"[All Fields]) OR (("sperm"[All Fields] AND "injections"[All Fields] AND "intracytoplasmic"[All Fields]) OR "intracytoplasmic sperm injections"[All Fields] OR ("intracytoplasmic"[All Fields] AND "sperm"[All Fields] AND "injections"[All Fields])) OR "icsi"[All Fields] OR ("artificial"[All Fields] AND "insemination"[All Fields]) OR "artificial insemination"[All Fields] OR (("ovulation"[MeSH Terms] OR "ovulation"[All Fields] OR "ovarian"[All Fields] OR "ovary"[MeSH Terms] OR "ovary"[All Fields]) AND ("induction"[All Fields] OR "stimulate"[All Fields] OR "stimulated"[All Fields] OR "stimulates"[All Fields] OR "stimulating"[All Fields] OR "stimulation"[All Fields] OR "stimulations"[All Fields] OR "stimulative"[All Fields] OR "stimulator"[All Fields] OR "stimulators"[All Fields] OR "hyperstimulated"[All Fields] OR "hyperstimulation"[All Fields] OR "hyperstimulations"[All Fields])) OR "ovulation induction"[MeSH Terms] OR "ovulation induction"[All Fields] OR "ovulation stimulation"[All Fields] OR "ovulation hyperstimulation"[All Fields] OR "ovarian induction"[All Fields] OR "ovarian stimulation"[All Fields] OR "ovarian hyperstimulation"[All Fields] OR "ovary induction"[All Fields] OR "ovary stimulation"[All Fields] OR "ovary hyperstimulation"[All Fields] OR ("controlled"[All Fields] AND ("ovarian"[All Fields] OR "ovary"[All Fields]) AND ("stimulate"[All Fields] OR "stimulated"[All Fields] OR "stimulates"[All Fields] OR "stimulating"[All Fields] OR "stimulation"[All Fields] OR "stimulations"[All Fields] OR "stimulative"[All Fields] OR "stimulator"[All Fields] OR "stimulators"[All Fields] OR "hyperstimulated"[All Fields] OR "hyperstimulation"[All Fields] OR "hyperstimulations"[All Fields])) OR "controlled ovarian hyperstimulation"[All Fields] OR "controlled ovary hyperstimulation"[All Fields] OR "controlled ovarian stimulation"[All Fields] OR "controlled ovary stimulation"[All Fields] OR "superovulation"[All Fields] OR "COS"[All Fields] OR "COH"[All Fields] OR (("oocyte"[All Fields] AND "retrieval"[All Fields]) OR "oocyte retrieval"[All Fields]) OR (("embryo"[All Fields] AND "transfer"[All Fields]) OR "embryo transfer"[All Fields] OR "IVF-ET"[All Fields])	
#3	(("gonadotropin-releasing hormone"[MeSH Terms] OR ("gonadotropin-releasing"[All Fields] AND "hormone"[All Fields]) OR "gonadotropin-releasing hormone"[All Fields] OR ("gonadotropin"[All Fields] AND "releasing"[All Fields] AND "hormone"[All Fields]) OR (("gonadotrophin"[All Fields] AND "releasing"[All Fields] AND "hormone"[All Fields]) OR "gonadotrophin releasing hormone"[All Fields]) OR "gonadoliberin"[All Fields] OR "gonadorelin"[All Fields] OR "luliberin"[All Fields] OR "LH-FSH releasing hormone"[All Fields] OR "LH-releasing hormone"[All Fields] OR "FSH-releasing hormone"[All Fields] OR "GnRH"[All Fields] OR "Gn-RH"[All Fields] OR "LHRH"[All Fields] OR "LH-RH"[All Fields] OR "LHFSHRH"[All Fields] OR "LFRH"[All Fields]) AND ("antagonists and inhibitors"[MeSH Subheading] OR ("antagonists"[All Fields] AND "inhibitors"[All Fields]) OR "antagonists and inhibitors"[All Fields] OR "antagonists"[All Fields] OR "antagonist"[All Fields] OR "inhibitors"[All Fields] OR "inhibitor"[All Fields])) OR "GnRH-ant"[All Fields] OR "GnRH-anta"[All Fields] OR "GnRHant"[All Fields] OR "GnRHanta"[All Fields] OR "GnRH ant"[All Fields] OR "GnRH anta"[All Fields] OR ("cetrorelix"[Supplementary Concept] OR "cetrorelix"[All Fields] OR "cetrolix"[All Fields] OR "cetrorelix acetate"[All Fields] OR "cetrorelix pamoate"[All Fields] OR "cetrotide"[All Fields]) OR ("abarelix"[Supplementary Concept] OR "abarelix"[All Fields] OR "plenaxis"[All Fields]) OR ("relugolix"[Supplementary Concept] OR "relugolix"[All Fields]) OR ("ganirelix"[Supplementary Concept] OR "ganirelix"[All Fields] OR "ganirelix acetate"[All Fields] OR "antagon"[All Fields] OR "orgalutran"[All Fields])	11,142
#4	(("gonadotropin-releasing hormone"[MeSH Terms] OR ("gonadotropin-releasing"[All Fields] AND "hormone"[All Fields]) OR "gonadotropin-releasing hormone"[All Fields] OR ("gonadotropin"[All Fields] AND "releasing"[All Fields] AND "hormone"[All Fields]) OR (("gonadotrophin"[All Fields] AND "releasing"[All Fields] AND "hormone"[All Fields]) OR "gonadotrophin releasing hormone"[All Fields]) OR "gonadoliberin"[All Fields] OR "gonadorelin"[All Fields] OR "luliberin"[All Fields] OR "LH-FSH releasing hormone"[All Fields] OR "LH-releasing hormone"[All Fields] OR "FSH-releasing hormone"[All Fields] OR "GnRH"[All Fields] OR "Gn-RH"[All Fields] OR "LHRH"[All Fields] OR "LH-RH"[All Fields] OR "LHFSHRH"[All Fields] OR "LFRH"[All Fields]) AND (("agonists"[Subheading] OR "agonists"[All Fields]) OR (agonist[All Fields]))) OR (GnRH-a[All Fields]) OR ("GnRHa"[All Fields]) OR "GnRH a"[All Fields] OR ("buserelin"[MeSH Terms] OR "buserelin"[All Fields] OR "busereline"[All Fields] ("buserelin"[All Fields] AND "acetate"[All Fields]) OR "buserelin acetate"[All Fields] "suprefact"[All Fields] OR "profact"[All Fields] OR "receptal"[All Fields] OR "tiloryth"[All Fields] OR "suprecur"[All Fields] OR "bigonist"[All Fields]) OR	16,240

	("goserelin"[MeSH Terms] OR "goserelin"[All Fields] OR gosereline[All Fields] OR ("goserelin"[All Fields] AND "acetate"[All Fields]) OR "goserelin acetate"[All Fields] OR "zoladex"[All Fields]) OR ("nafarelin"[MeSH Terms] OR "nafarelin"[All Fields] OR nafareline[All Fields] OR ("nafarelin"[All Fields] AND "acetate"[All Fields]) OR "nafarelin acetate"[All Fields] OR "synarel"[All Fields]) OR (triptoielin[All Fields] OR "triptorelin pamoate"[MeSH Terms] OR ("triptorelin"[All Fields] AND "pamoate"[All Fields]) OR "triptorelin pamoate"[All Fields] OR "triptoreline"[All Fields] OR triptrolein[All Fields] OR "triptorelyn"[All Fields] OR "decapeptyl"[All Fields]) OR ("leuprolide"[MeSH Terms] OR "leuprolide"[All Fields] OR "leuprorelin"[All Fields] OR leuprolin[All Fields] OR ("leuprorelin"[All Fields] AND "acetate"[All Fields]) OR "leuprorelin acetate"[All Fields] OR ("leuprolide"[All Fields] AND "acetate"[All Fields]) OR "leuprolide acetate"[All Fields] OR "enantone"[All Fields] OR "lupron"[All Fields])	
#5	"randomized controlled trial"[Publication Type] OR "randomized controlled trials as topic"[MeSH Terms] OR "randomized controlled trial"[All Fields] OR "randomised controlled trial"[All Fields] OR ("clinical trial"[Publication Type] OR "clinical trials as topic"[MeSH Terms] OR "clinical trial"[All Fields] OR "intervention study"[All Fields]) OR ("controlled"[All Fields] AND ("clinical trials as topic"[MeSH Terms] OR "clinical trial"[All Fields] OR "trial"[All Fields] OR "trials"[All Fields])) OR "RCT"[All Fields] OR "randomized clinical trial"[All Fields] OR "controlled trial"[All Fields] OR "controlled clinical trial"[All Fields] OR "randomized trial"[All Fields] OR "randomized trials"[All Fields] OR "randomized study"[All Fields] OR "randomized studies"[All Fields] OR "randomized prospective study"[All Fields] OR "randomized prospective studies"[All Fields] OR "randomized prospective trial"[All Fields] OR "randomized prospective trials"[All Fields] OR "random allocation"[All Fields] OR "random assignment"[All Fields] OR ("random"[All Fields] OR "randomization"[All Fields] OR "randomized"[All Fields] OR "randomisation"[All Fields] OR "randomisations"[All Fields] OR "randomise"[All Fields] OR "randomised"[All Fields] OR "randomising"[All Fields] OR "randomizations"[All Fields] OR "randomize"[All Fields] OR "randomizes"[All Fields] OR "randomizing"[All Fields] OR "randomness"[All Fields] OR "randoms"[All Fields]) OR (("random"[All Fields] OR "randomization"[All Fields] OR "randomized"[All Fields] OR "randomisation"[All Fields] OR "randomisations"[All Fields] OR "randomise"[All Fields] OR "randomised"[All Fields] OR "randomising"[All Fields] OR "randomizations"[All Fields] OR "randomize"[All Fields] OR "randomizes"[All Fields] OR "randomizing"[All Fields] OR "randomness"[All Fields] OR "randoms"[All Fields]) AND ("controlled trial"[All Fields] OR ("controlled"[All Fields] AND "trial"[All Fields]) OR "clinical trials as topic"[MeSH Terms] OR "clinical trials as topic"[All Fields] OR "trial"[All Fields] OR "trials"[All Fields] OR "study"[All Fields] OR "studies"[All Fields] OR "prospective studies"[MeSH Terms] OR ("prospective"[All Fields] AND "studies"[All Fields]) OR "prospective studies"[All Fields] OR ("prospective"[All Fields] AND "study"[All Fields]) OR "prospective study"[All Fields] OR ("prospective"[All Fields] AND "trials"[All Fields]) OR "prospective trials"[All Fields] OR ("prospective"[All Fields] AND "trial"[All Fields]) OR "prospective trial"[All Fields] OR "allocation"[All Fields] OR ("assign"[All Fields] OR "assignation"[All Fields] OR "assignations"[All Fields] OR "assigned"[All Fields] OR "assigning"[All Fields] OR "assignment"[All Fields] OR "assignments"[All Fields] OR "assigns"[All Fields])))	1,782,009
#5<br><br>PCOS/RCT	#1 AND #2 AND #3 AND #4 AND #5	57

SCOPUS Database

#1	TITLE-ABS-KEY("polycystic ovar* syndrome" OR ("polycystic" AND "ovar*" AND "syndrome") OR "polycystic ovar* disease" OR ("polycystic" AND "ovar*" AND " disease") OR "Stein Leventhal syndrome" OR ("Stein Leventhal" AND "syndrome") OR "sclerocystic ovar* syndrome" OR ("sclerocystic" AND "ovar*" AND "syndrome") OR "sclerocystic ovar* degeneration" OR ("sclerocystic" AND "ovar*" AND "degeneration") OR "PCOS" OR "PCOD" OR "PCO")	37,706
#2	TITLE-ABS-KEY("assisted reproducti* techn*" OR ("assisted" AND "reproducti*" AND "techn*") OR "reproducti* medic*" OR ("reproducti*" AND "medic*") OR "fertili?ation in vitro" OR ("fertili?ation"	394,721

	AND "in vitro") OR "IVF" OR "test-tube fertili?ation" OR ("test-tube" AND "fertili?ation") OR "test-tube baby" OR ("test-tube" AND "baby") OR "IVF-ET" OR "intracytoplasmic sperm injections" OR ("intracytoplasmic" AND "sperm" AND "injections") OR "ICSI" OR "artificial insemination" OR ("artificial" AND "insemination") OR "ovulat* induc*" OR "ovulat* stimulat*" OR "ovulat* hyperstimulat*" OR "ovar* induc*" OR "ovar* stimulat*" OR "ovar* hyperstimulat*" OR (("ovulat*" OR "ovar*") AND (" induc*" OR "stimulat*" OR "hyperstimulat*")) OR "control* ovar* stimulat*" OR "control* ovar* hyperstimulat*" OR ("control*" AND "ovar*" AND ("stimulat*" OR "hyperstimulat*")) OR "superovulat*" OR "COS" OR "COH" OR "oocyte* retrieval$" OR ("oocyte*" AND "retrieval$") OR "embryo* transfer*" OR ("embryo*" AND "transfer*"))	
#3	TITLE-ABS-KEY((("gonadotrop?in-releasing hormone" OR ("gonadotrop?in-releasing" AND "hormone") OR ("gonadotrop?in" AND "releasing" AND "hormone") OR "Gonadoliberin" OR "Gonadorelin" OR "Luliberin" OR "LH-FSH Releasing Hormone" OR "LH Releasing Hormone" OR "FSH Releasing Hormone" OR "GNRH" OR "GN-RH" OR "LHRH" OR "LH-RH" OR "LHFSHRH" OR "LFRH") AND ("antagonist" OR " inhibitor")) OR "GnRH-ant" OR "GnRH-anta" OR "GnRHant" OR "GnRHanta" OR "GnRH ant" OR "GnRH anta" OR "cetrorelix" OR "cetrolix" OR "cetrorelix acetate" OR "cetrorelix pamoate" OR "cetrotide" OR "abarelix" OR "plenaxis" OR "relugolix" OR "ganirelix" OR "ganirelix acetate" OR "antagon" OR "orgalutran")	17,111
#4	TITLE-ABS-KEY((("gonadotrop?in-releasing hormone" OR ("gonadotrop?in-releasing" AND "hormone") OR ("gonadotrop?in" AND "releasing" AND "hormone") OR "Gonadoliberin" OR "Gonadorelin" OR "Luliberin" OR "LH-FSH Releasing Hormone" OR "LH Releasing Hormone" OR "FSH Releasing Hormone" OR "GNRH" OR "GN-RH" OR "LHRH" OR "LH-RH" OR "LHFSHRH" OR "LFRH") AND ("agonist")) OR "GnRH-a" OR "GnRHa" OR "GnRH a" OR "buserelin" OR "busereline" OR "buserelin acetate" OR "suprefact" OR "profact" OR "receptal" OR "tiloryth" OR "suprecur" OR "bigonist" OR "goserelin" OR "gosereline " OR "goserelin acetate" OR " Zoladex" OR "nafarelin" OR "nafareline" OR "nafarelin acetate" OR "Synarel" OR "triptoielin" OR "triptoreline" OR "triptorelin pamoate" OR "triptorelyn" OR "triptrolein" OR "Decapeptyl" OR "leuprorelin" OR "leuprolin" OR "leuprorelin acetate" OR "leuprolide" OR "leuprolide acetate" OR "Enantone" OR "Lupron")	34,999
#5	TITLE-ABS-KEY("random* control* trial*" OR "random* clinical trial*" OR "clinical trial*" OR "control* trial*" OR "control* clinical trial*" OR ("control*" AND ("clinical trial*" OR "trial*")) OR "random* trial*" OR "RCT" OR "random* stud*" OR " random* prospective stud*" OR " random* prospective trial*" OR "intervention stud*" OR "random*" OR "random* allocat*" OR "random* assign*" OR ("random*" AND ("control* trial*" OR ("control*" AND "trial*") OR "clinical trial*" OR "trial*" OR "stud*" OR ("prospective" AND "stud*") OR "prospective stud*" OR ("prospective" AND " trial*") OR "prospective trial*" OR "allocat*" OR "assign*")))	3,750,781
#6<br>PCOS/RCT	#1 AND #2 AND #3 AND #4 AND #5	**162**

Web Of Science

#1	TS=("polycystic ovar* syndrome" OR ("polycystic" AND "ovar*" AND "syndrome") OR "polycystic ovar* disease" OR ("polycystic" AND "ovar*" AND " disease") OR "Stein Leventhal syndrome" OR ("Stein Leventhal" AND "syndrome") OR "sclerocystic ovar* syndrome" OR ("sclerocystic" AND "ovar*" AND "syndrome") OR "sclerocystic ovar* degeneration" OR ("sclerocystic" AND "ovar*" AND "degeneration") OR "PCOS" OR "PCOD" OR “PCO”)	34,412
#2	TS=("assisted reproducti* techn*" OR ("assisted" AND "reproducti*" AND "techn*") OR "reproducti* medic*" OR ("reproducti*" AND "medic*") OR "fertili?ation in vitro" OR ("fertili?ation" AND "in vitro") OR "IVF" OR "test-tube fertili?ation" OR ("test-tube" AND "fertili?ation") OR "test-tube baby" OR ("test-tube" AND "baby") OR "IVF-ET" OR "intracytoplasmic sperm injections" OR ("intracytoplasmic" AND "sperm" AND "injections") OR "ICSI" OR "artificial insemination" OR	249,933

	("artificial" AND "insemination") OR "ovulat* induc*" OR "ovulat* stimulat*" OR "ovulat* hyperstimulat*" OR "ovar* induc*" OR "ovar* stimulat*" OR "ovar* hyperstimulat*" OR (("ovulat*" OR "ovar*") AND (" induc*" OR "stimulat*" OR "hyperstimulat*")) OR "control* ovar* stimulat*" OR "control* ovar* hyperstimulat*" OR ("control*" AND "ovar*" AND ("stimulat*" OR "hyperstimulat*")) OR "superovulat*" OR "COS" OR "COH" OR "oocyte* retrieval$" OR ("oocyte*" AND "retrieval$") OR "embryo* transfer*" OR ("embryo*" AND "transfer*"))	
#3	TS=((("gonadotrop?in-releasing hormone" OR ("gonadotrop?in-releasing" AND "hormone") OR ("gonadotrop?in" AND "releasing" AND "hormone") OR "Gonadoliberin" OR "Gonadorelin" OR "Luliberin" OR "LH-FSH Releasing Hormone" OR "LH Releasing Hormone" OR "FSH Releasing Hormone" OR "GNRH" OR "GN-RH" OR "LHRH" OR "LH-RH" OR "LHFSHRH" OR "LFRH") AND ("antagonist*" OR " inhibitor*")) OR "GnRH-ant" OR "GnRH-anta" OR "GnRHant" OR "GnRHanta" OR "GnRH ant" OR "GnRH anta" OR "cetrorelix" OR "cetrolix" OR "cetrorelix acetate" OR "cetrorelix pamoate" OR "cetrotide" OR "abarelix" OR "plenaxis" OR "relugolix" OR "ganirelix" OR "ganirelix acetate" OR "antagon" OR "orgalutran")	8,865
#4	TS=((("gonadotrop?in-releasing hormone" OR ("gonadotrop?in-releasing" AND "hormone") OR ("gonadotrop?in" AND "releasing" AND "hormone") OR "Gonadoliberin" OR "Gonadorelin" OR "Luliberin" OR "LH-FSH Releasing Hormone" OR "LH Releasing Hormone" OR "FSH Releasing Hormone" OR "GNRH" OR "GN-RH" OR "LHRH" OR "LH-RH" OR "LHFSHRH" OR "LFRH") AND ("agonist*")) OR "GnRH-a" OR "GnRHa" OR "GnRH a" OR "buserelin" OR "busereline" OR "buserelin acetate" OR "suprefact" OR "profact" OR "receptal" OR "tiloryth" OR "suprecur" OR "bigonist" OR "goserelin" OR "gosereline " OR "goserelin acetate" OR " Zoladex" OR "nafarelin" OR "nafareline" OR "nafarelin acetate" OR "Synarel" OR "triptoielin" OR "triptoreline" OR "triptorelin pamoate" OR "triptorelyn" OR "triptrolein" OR "Decapeptyl" OR "leuprorelin" OR "leuprolin" OR "leuprorelin acetate" OR "leuprolide" OR "leuprolide acetate" OR "Enantone" OR "Lupron")	15,892
#5	TS=("random* control* trial*" OR "random* clinical trial*" OR "clinical trial*" OR "control* trial*" OR "control* clinical trial*" OR ("control*" AND ("clinical trial*" OR "trial*")) OR "random* trial*" OR "RCT" OR "random* stud*" OR " random* prospective stud*" OR " random* prospective trial*" OR "intervention stud*" OR "random*" OR "random* allocat*" OR "random* assign*" OR ("random*" AND ("control* trial*" OR ("control*" AND "trial*") OR "clinical trial*" OR "trial*" OR "stud*" OR ("prospective" AND "stud*") OR "prospective stud*" OR ("prospective" AND " trial*") OR "prospective trial*" OR "allocat*" OR "assign*")))	2,397,398
#6<br><br>PCOS/RCT	#1 AND #2 AND #3 AND #4 AND #5	**51**

CINAHL Database

S1	(MM "Polycystic Ovary Syndrome")	3,097
S2	TX polycystic N3 ovar*	6,269
S3	TX stein-leventhal syndrome	26
S4	TX ovar* N1 (scelerocystic or degeneration)	14
S5	TX PCOS OR TX PCOD OR TX PCO	4,688
**S6**	S1 OR S2 OR S3 OR S4 OR S5	7,968
S7	(MM "Reproduction Techniques+")	10,283
S8	TX assisted reproducti* techn*	4,541
S9	TX reproducti* medic*	18,628

S10	(MM "Fertilization in Vitro")	3,903
S11	TX vitro fertili?ation	9,964
S12	TX test-tube fertili?ation	19
S13	TX test-tube baby	143
S14	TX IVF OR TX IVF-ET OR TX ICSI	8,510
S15	TX intracytoplasmic sperm injection*	1,442
S16	(MM "Insemination, Artificial")	521
S17	TX artificial insemination	1,299
S18	TX ovulat* N3 (induc* or stimulat* or hyperstimulat*)	2,480
S19	TX ovar* N3 (induc* or stimulat* or hyperstimulat*)	4,318
S20	TX control* N3 ovar* N3 (stimulat* or hyperstimulat*)	697
S21	TX superovulat*	184
S22	TX COH or TX COS	5,088
S23	TX oocyte* retrieval$	681
S24	(MM "Embryo Transfer")	1,297
S25	TX embryo* transfer*	4,507
**S26**	S7 OR S8 OR S9 OR S10 OR S11 OR S12 OR S13 OR S14 OR S15 OR S16 OR S17 OR S18 OR S19 OR S20 OR S21 OR S22 OR S23 OR S24 OR S25	42,582
S27	(MM "Gonadorelin+")	1,287
S28	TX gonadotrop?in releasing hormone	282
S29	TX (gonadorelin or luliberin or Gonadoliberin)	1,887
S30	TX (“LH-FSH Releasing Hormone” OR "LH Releasing Hormone" OR "FSH Releasing Hormone")	16
S31	TX (GnRH or GN-RH or LHRH or LH-RH or LHFSHRH or LFRH)	2,931
S32	S27 OR S28 OR S29 OR S30 OR S31	4,363
S33	TX (antagonist* OR inhibitor*)	252,716
**S34**	S32 AND S33	2,090
S35	TX (GnRH-ant OR GnRH-anta OR GnRHant OR GnRHanta OR GnRH ant OR GnRH anta)	14
S36	TX (cetrorelix OR cetrolix OR cetrorelix acetate OR cetrorelix pamoate OR cetrotide)	71
S37	TX (abarelix OR plenaxis OR relugolix)	92
S38	TX (ganirelix OR ganirelix acetate OR antagon OR orgalutran)	78
**S39**	S34 OR S35 OR S36 OR S37 OR S38	2,187
S40	TX agonist*	36,911
**S41**	S32 AND S40	2,007
S42	TX GnRH-a OR TX GnRHa OR TX GnRH a	2,444

S43	TX buserelin OR TX busereline OR TX buserelin acetate OR TX suprefact OR TX profact OR TX receptal OR TX tiloryth OR TX suprecur OR TX bigonist	97
S44	TX goserelin OR TX gosereline OR TX goserelin acetate OR TX Zoladex	754
S45	TX nafarelin OR TX nafareline OR TX nafarelin acetate OR TX Synarel	81
S46	TX triptoielin OR TX triptoreline OR TX triptorelin pamoate OR TX triptorelyn OR TX triptrolein OR TX Decapeptyl	73
S47	TX leuprorelin OR TX leuprolin OR TX leuprorelin acetate OR TX leuprolide OR TX leuprolide acetate OR TX Enantone OR TX Lupron	1,141
**S48**	S41 OR S42 OR S43 OR S44 OR S45 OR S46 OR S47	4,256
S49	(MH "Clinical Trials+")	309,635
S50	PT Clinical trial	108,302
S51	TX clinic* n1 trial*	358,638
S52	TX random* control* trial*	274,419
S53	TX random* clinical trial*	63,364
S54	TX control* trial*	299,347
S55	TX "control* clinical trial*"	18,411
S56	TX random* trial*	342,378
S57	TX RCT*	35,504
S58	TX random* stud*	146,937
S59	TX random* prospective stud*	22,562
S60	TX random* prospective trial*	22,744
S61	TX intervention stud*	95,588
S62	TX random*	602,149
S63	(MH "Random Assignment")	64,787
S64	TX allocat* random*	19,485
S65	TX random* allocat*	19,485
**S66**	S49 OR S50 OR S51 OR S52 OR S53 OR S54 OR S55 OR S56 OR S57 OR S58 OR S59 OR S60 OR S61 OR S62 OR S63 OR S64 OR S65	838,030
**S68** PCOS/RCT	S6 AND S26 AND S39 AND S48 AND S66	61

TRIP Database

+ Controlled trials filter	("polycystic ovary syndrome" OR "polycystic ovarian syndrome" OR "polycystic ovary disease" OR "polycystic ovarian disease" OR "Stein Leventhal syndrome" OR "sclerocystic ovary syndrome" OR "PCOS" OR "PCOD" OR "PCO") AND ("assisted reproductive techniques" OR "reproductive medicine" OR "in vitro fertilization" OR "fertilization in vitro" OR "in vitro fertilisation" OR "fertilisation in vitro" OR "IVF" OR "intracytoplasmic sperm injections" OR "ICSI" OR "artificial insemination" OR (("ovulation" OR "ovarian") AND ("stimulation" OR "hyperstimulation"))) AND ((("gonadotropin	39

	releasing hormone" OR "gonadotrophin releasing hormone" OR "GNRH" OR "GN-RH" OR "LH-RH") AND ("antagonists" OR " inhibitors")) OR "gnrhant" OR "gnrhanta" OR "gnrh ant" OR "gnrh anta" OR "cetrorelix" OR "cetrolix" OR "cetrotide" OR "abarelix" OR "plenaxis" OR "relugolix" OR "ganirelix" OR "antagon" OR "orgalutran") AND ((("gonadotropin releasing hormone" OR "gonadotrophin releasing hormone" OR "GNRH" OR "GN-RH" OR "LH-RH") AND ("agonists")) OR "gnrh-a" OR "gnrha" OR "gnrh a" OR "buserelin" OR "Suprefact" OR "Profact" OR "goserelin" OR " Zoladex" OR "nafarelin" OR "Synarel" OR "triptorelin" OR "Decapeptyl" OR "leuprolide" OR "Lupron" OR "Enantone")	

Clinicaltrials.gov (15)

Condition: "polycystic ovary syndrome" OR "PCOS" OR "PCOD"

Other terms: (IVF OR ICSI) AND (gonadotropin releasing hormone Agonists OR GNRH Agonists) AND (gonadotropin releasing hormone Antagonists OR GNRH Antagonists)

ISRCTN registry (2)

Condition: PCOS, Intervention: IVF

Condition: PCOS, Intervention: ICSI

Supplementary Table S4: Studies characteristics:

Bahçeci et al., 2005

**Methods**	**Study design:** RCT, single-center, parallel design, **Study duration:** Nov 2001– Nov 2002, **Country:** Turkey	
**Participants**	**Inclusion criteria:** PCOS defined as primary infertility, oligomenorrhoea, clinical hyperandrogenism (hirsutism Ferriman-Galwey score > 7), Reversed FSH/LH ratio and polycystic appearance of the ovaries on ultrasound.	
	**Exclusion criteria:** women who had undergone previous ART or had hyperprolactinaemia or thyroid abnormalities. Couples with coexisting male factor infertility due to nonobstructive azoospermia.	
	**Baseline characteristics:**	**GnRH Antagonist group: Age:** 30.06±4.8 years. **BMI:** 26.1±3.8 Kg/m^2. **Infertility Duration:** 5.15±2.2 years. **FSH:** 5.05±2.2 mIU/ml. **LH:** 9.68±4.3 mIU/ml. **AMH:** NI.
		**GnRH Agonist group: Age:** 29.43±4.3 years. **BMI:** 26.03±4.2 Kg/m^2. **Infertility Duration:** 4.73±2.6 years. **FSH:** 4.77±1.6 mIU/ml. **LH:** 8.25±4.06 mIU/ml. **AMH:** NI.
	**Group size**	**GnRH Antagonist group:** 73 women were allocated, 59 women were analyzed.
		**GnRH Agonist group:** 75 women were allocated, 70 women were analyzed.
	**Lost to follow up/ drop-out**	**GnRH Antagonist group:** 14 women dropped out before starting ovulation induction for personal reasons.
		**GnRH Agonist group:** 5 women dropped before starting ovulation induction for personal reasons.
**Intervention**	**GnRH Antagonist:** Flexible; Cetrorelix (the lead follicle reached 14 mm). **Pretreatment:** OCP for 21 days from the preceding menstrual cycle (ethinyl estradiol 0.03 mg + gestoden 0.075 mg; Ginera, Schering, Istanbul, Turkey). **Stimulator:** FSH/hMG, D3. **Stimulator Starting Dose:** 150-225 IU. **Trigger:** 10,000 IU hCG (≥ 2 follicles reached 18 mm). **Luteal support:** 100 mg/day progesterone in oil, IM. **Transfer day:** Day 3.	
**Comparison**	**GnRH Agonist:** Long; Leuprolide from D14 of the menstrual cycle. **Pretreatment:** OCP for 21 days from the preceding menstrual cycle (ethinyl estradiol 0.03 mg + gestoden 0.075 mg; Ginera, Schering, Istanbul, Turkey). **Stimulator:** FSH/hMG, D3. **Stimulator Starting Dose:** 150-225 IU. **Trigger:** 10,000 IU hCG (≥ 2 follicles reached 18 mm). **Luteal support:** 100 mg/day progesterone in oil, IM. **Transfer day:** Day 3.	
**Notes**	**Randomization:** using a table of random numbers. **Allocation:** NI. **Baseline imbalances:** NO. **ITT/mITT or PP:** mITT. **Blinded Participates:** NI. **Blinded intervention providers:** NI. **Blinded outcomes assessors:** NI. **Funding:** NI. **OHSS criteria:** Not reported. **Cycle cancellation criteria:** Not reported, all women underwent ET, No cycle cancellation.	
**Study records**	**Bahçeci et al., 2005** [2]**.**	

Choi et al., 2005

**Methods**	**Study design:** RCT, single-center, parallel design. **Study duration:** June 2000-February 2004. **Country:** Korea.	
**Participants**	**Inclusion criteria:** PCOS women diagnosed based on Rotterdam criteria.	
	**Exclusion criteria:** Women with a history of other hormonal therapy during the 3 months before the hyperstimulation. Women with a history of internal or surgical diseases could affect the study results.	
	**Baseline characteristics:**	**GnRH Antagonist group: Age:** 32.8±3.4 years. **BMI:** 25.6±3.2 Kg/m^2. **Infertility Duration:** 3.2±1.5 years. **FSH:** 5.2±1.4 mIU/ml. **LH:** 6.9±1.8 mIU/ml. **AMH:** NI.
		**GnRH Agonist group: Age:** 32.0±3.6 years. **BMI:** 25.7±3.4 Kg/m^2. **Infertility Duration:** 3.6±1.7 years. **FSH:** 5.8±1.4 mIU/ml. **LH:** 7.3±2.1 mIU/ml. **AMH:** NI.
	**Group size**	**GnRH Antagonist group:** 22 women.
		**GnRH Agonist group:** 21 women.
	**Lost to follow up/ drop-out**	**GnRH Antagonist group:** None.
		**GnRH Agonist group:** None.
**Intervention**	**GnRH Antagonist:** Flexible; Cetrorelix (lead follicle reached ≥ 13 mm). **Pretreatment:** OCP for 21 days from the preceding menstrual period (ethinyl estradiol 0.035 mg+cyproterone acetate 2 mg, Diane 35, Schering AG, Germany).	

	**Stimulator:** r-FSH, D3. **Stimulator Starting Dose:** NI. **Trigger:** 5000-10000 IU hCG (≥ 1 follicle with diameter ≥ 18 mm or ≥ 2 follicles with diameter > 17 mm). **Luteal support:** 50 mg Progesterone in oil from the day of oocyte retrieval. **Transfer day:** Day 3.
**Comparison**	**GnRH Agonist:** Long; Triptorelin from D20 of menstural cycle. **Pretreatment:** OCP for 21 days from the preceding menstrual period (ethinyl estradiol 0.035 mg+cyproterone acetate 2 mg, Diane 35, Schering AG, Germany). **Stimulator:** r-FSH, D3. **Stimulator Starting Dose:** NI. **Trigger:** 5000-10000 IU hCG (≥ 1 follicle with diameter ≥ 18 mm or ≥ 2 follicles with diameter > 17 mm). **Luteal support:** 50 mg Progesterone in oil from the day of oocyte retrieval. **Transfer day:** Day 3.
**Notes**	**Randomization:** Randomized, no further information. **Allocation:** NI. **Baseline imbalances:** NO. **ITT/mITT or PP:** ITT/mITT. **Blinded Participates:** NI. **Blinded intervention providers:** NI. **Blinded outcomes assessors:** NI. **Funding:** NI. **OHSS criteria:** Not reported. **Cycle cancellation criteria:** Not reported.
**Study records**	**Choi et al., 2005** [3]**.**

Ghaebi et al., 2018

**Methods**	**Study design:** RCT, single-center, parallel design. **Study duration:** Mar 2013 -Sep 2015. **Country:** Iran.	
**Participants**	**Inclusion criteria:** PCOS aged 18-37 years, basal FSH <10, normal thyroid and prolactin tests, normal uterine cavity, with or without tubal obstruction and without hydrosalpinx (based on hysterosalpingography or hysteroscopy), with or without male factor infertility (according to world health organization criteria). PCOS diagnosis was based on AES, 2006 criteria.	
	**Exclusion criteria:** Women with tubal obstruction with hydrosalpinx, poor response or low response in previous IVF cycles, congenital uterine abnormalities, heart, liver, kidney, ovarian cancer, women aged > 38 years.	
	**Baseline characteristics:**	**GnRH Antagonist group: Age:** 29.43±4.34 years. **BMI:** NI. **Infertility Duration:** Primary infertility: 94.24±52.81 months, Secondary infertility: 35.25±40.34 months. **FSH:** 5.94±3.11 mIU/ml. **LH:** 7.1±3.99 mIU/ml. **AMH:** NI.
		**GnRH Agonist group: Age:** 31±4.43 years. **BMI:** NI. **Infertility Duration:** Primary infertility: 78.09±44.6 months, Secondary infertility: 35.25±20.39 months. **FSH:** 7.28±3.27 mIU/ml. **LH:** 6.48±4.4 mIU/ml. **AMH:** NI.
	**Group size**	**GnRH Antagonist group:** 50 women were randomized, 46 women were analyzed.
		**GnRH Agonist group:** 50 were randomized, 50 women were analyzed.
	**Lost to follow up/ drop-out**	**GnRH Antagonist group:** 4 women drop out, no reasons were reported.
		**GnRH Agonist group:** None.
**Intervention**	**GnRH Antagonist:** Flexible; Cetrorelix (the lead follicle reached 12-14 mm). **Pretreatment:** OCP started from Day5 of the menstrual cycle, and for day 21 of the cycle. **Stimulator:** r-FSH; D2. **Stimulator Starting Dose:** 150 IU. **Trigger:** 10000 IU hCG (2-3 follicles reached 17 mm). **Luteal support:** NI. **Transfer day:** Day 2-3.	
**Comparison**	**GnRH Agonist:** Long; Buserelin from D21 of the cycle. **Pretreatment:** OCP started from Day5 of the menstrual cycle, and for day 21 of the cycle. **Stimulator:** r-FSH; when the criteria of downregulation were met; If serum estradiol level < 50 pg/ml and LH level < 5 mU/ml and endometrial thickness < 5 mm in vaginal ultrasound. **Stimulator Starting Dose:** 150 IU. **Trigger:** 5000- 10000 IU hCG (2-3 follicles reached 17-18 mm). **Luteal support:** NI. **Transfer day:** Day 2-3.	
**Notes**	**Randomization:** No information about how randomized sequence was generated. **Allocation:** Randomization was done by an independent person using sealed envelopes. **Baseline imbalances:** NO. **ITT/mITT or PP:** mITT. **Blinded participates:** NI. **Blinded intervention providers:** NI. **Blinded outcomes assessors:** NI. **Funding:** NI. **OHSS criteria:** reported, patients with clinical signs of abdominal pain, distension, nausea and vomiting, and oliguria were considered as ovarian hyperstimulation syndrome. **Cycle cancellation criteria:** reported, In case of observing clinical symptoms of OHSS 3 days after OPU or observing more than 10 small (< 12 mm) or medium (12-14 mm) follicles or more than 15 large follicles the day before hCG administration, embryo transfer was not performed on patients, but they did not mention whether they would have noted any cycle cancellation cases in the study's groups.	

**Study records**	**Ghaebi et al., 2018** [4]**.**

Haydardedeoglu et al., 2012

**Methods**	**Study design:** RCT, single-center, parallel design. **Study duration:** Mar 2009- Jun 2011. **Country:** Turkey.	
**Participants**	**Inclusion criteria:** PCOS women in their first IVF/ICSI cycles, aged <35 years and >23 years, whose BMI <30 Kg/m^2 and >20 Kg/m^2. PCOS diagnosis was based on Rotterdam criteria; all women with oligomenorrhea (an irregular cycle duration greater than 45 days or less than 6 menstrual periods per year) and/or anovulation who also had at least one of the characteristics of hyperandrogenism (a hirsutism score of greater than 7 according to Ferriman and Gallwey 1961, and/or an elevated serum testosterone level which is over 0.8 ng/dl after excluding all the other causes of hyperandrogenism. All participants have polycystic ovaries identified by ultrasonography as the presence of 12 or more follicles in each ovary measuring 2 – 9 mm in diameter, and/or increased ovarian volume (> 10 ml).	
	**Exclusion criteria:** Women with PCOS whose ovaries did not appear polycystic (identified by ultrasonography was defined as the presence of 12 or more follicles in each ovary measuring 2 – 9 mm in diameter, and/or increased ovarian volume (> 10 ml)). Women treated with hormonal medications and other oral anti-diabetics within the previous three months.	
	**Baseline characteristics:**	**GnRH Antagonist group: Age:** 27.57 ± 3.54 years. **BMI:** 25.74 ± 4.37 Kg/m^2. **Infertility Duration:** 6.24 ± 3.64 years. **FSH:** 4.77 ± 1.80 mIU/ml. **LH:** 5.94 ± 4.17 mIU/ml. **AMH:** NI.
		**GnRH Agonist group: Age:** 27.70 ± 3.59 years. **BMI:** 24.97 ± 4.36Kg/m^2. **Infertility Duration:** 5.85 ± 3.42 years. **FSH:** 5.03 ± 1.36 mIU/ml. **LH:** 5.60 ± 3.49 mIU/ml. **AMH:** NI.
	**Group size**	**GnRH Antagonist group:** 150 women.
		**GnRH Agonist group:** 150 women.
	**Lost to follow up/ drop-out**	**GnRH Antagonist group:** None.
		**GnRH Agonist group:** None.
**Intervention**	**GnRH Antagonist:** Fixed; Ganirelix from S6. **Pretreatment:** OCP from Day3 of the preceding menstrual cycle, for 21 days (ethinyl estradiol 0.03 mg+drosprinone 3 mg, Yasmin, Schering, Istanbul, Turkey). **Stimulator:** r-FSH, D3. **Stimulator Starting Dose:** 150 IU. **Trigger:** 10,000 IU hCG (≥ 3 follicles having a maximum diameter >17 mm). **Luteal support:** 90 mg/day progesterone intra-vaginally starting after ET. **Transfer day:** Day 3.	
**Comparison**	**GnRH Agonist:** Long; Leuprolide from D21of the preceding cycle. **Pretreatment:** OCP from Day3 of the preceding menstrual cycle, for 21 days (ethinyl estradiol 0.03 mg+drosprinone 3 mg, Yasmin, Schering, Istanbul, Turkey). **Stimulator:** r-FSH, if there were no cysts ≥ 2 cm and the E2 was <50 pg/ml. **Stimulator Starting Dose:** 150 IU. **Trigger:** 10,000 IU hCG (≥ 3 follicles having a maximum diameter >17 mm). **Luteal support:** 90 mg/day progesterone intra-vaginally starting after ET. **Embryo transfer day:** Day 3.	
**Notes**	**Randomization**: using a random numbers table in blocks of 30. **Allocation:** using consecutively numbered opaque, sealed envelopes on the day of initiation of OCP. The envelopes were opened by the ART nurse coordinator who had no other involvement in the trial. **Baseline imbalances:** NO. **ITT/mITT or PP:** ITT/mITT. **Blinded participates:** PN, (Registry record). **Blinded intervention providers:** PY, (Registry record). **Blinded outcomes assessors:** PY, (Registry record). **Funding:** unclear. **OHSS criteria:** hospitalized OHSS was diagnosed when the hematocrit level rose above 45 % and abdominal discomfort, and/or progressive oliguria and/or respiratory difficulties were found together with moderate ascites and/or thrombocytosis (platelet count > 400,000/ l), and leucocytosis (white blood cell count > 12,000/ l). **Cycle cancellation criteria:** The cycle was cancelled if there was monofollicular development (single dominant follicle over 17 mm) and/or serum progesterone level was >1.5 ng/ml on the day of hCG). Likewise, women deemed under high risk of OHSS based on the number of growing follicles or high serum E2 levels, and women who had abruptly decreasing E2 levels during coasting and couples with total fertilization failure had their cycles cancelled.	
**Study records**	**Haydardedeoglu et al., 2012** [5]**, a registry record (NCT01354275)** [6]**.**	

Hosseini et al., 2010

**Methods**	**Study design:** RCT, single-center, parallel design. **Study duration:** 2006. **Country:** Iran.

**Participants**	**Inclusion criteria:** PCOS, < 35 years, normal BMI (< 27 kg/m^2), normal prolactin, normal thyroid levels, normal spermogram. PCOS diagnosis was based on Rotterdam criteria; the presence of two of the three following characteristics for inclusion in the study:<br>(i) oligomenorrhea/amenorrhea.<br>(ii) clinical finding of hyperandrogenism (they did not measure androgens for chemical hyperandrogenism, hirsutism was accepted as a clinical finding of hyperandrogenism).<br>(iii) polycystic ovaries on transvaginal sonography.	
	**Exclusion criteria:** NI.	
	**Baseline characteristics:**	**GnRH Antagonist group: Age:** 27.75 ± 3.40 years. **BMI:** all participants BMI (< 27 kg/m^2). **Infertility Duration:** 2.82 ± 0.8 years. **FSH:** 5.65 ± 2.41 mIU/ml. **LH:** 8.06 ± 4.43 mIU/ml. **AMH:** NI.
		**GnRH Agonist group: Age:** 29.31 ± 4.23 years. **BMI:** all participants BMI (< 27 kg/m^2). **Infertility Duration:** 2.87 ± 1 years. **FSH:** 6.24 ± 4.44 mIU/ml. **LH:** 7.50 ± 3.76 mIU/ml. **AMH:** NI.
	**Group size**	**GnRH Antagonist group:** 57 women.
		**GnRH Agonist group:** 55 women.
	**Lost to follow up/ drop-out**	**GnRH Antagonist group:** None.
		**GnRH Agonist group:** None.
**Intervention**	**GnRH Antagonist:** Flexible; Cetrorelix (a follicle reached 14 mm). **Pretreatment:** folic acid 1 mg/day before initiation of the induction cycle + low dose OCP on day3 of the previous cycle + doxycycline 100 mg twice daily for the first 10 days of the previous cycle. **Stimulator:** r-FSH, D3 + hMG was prescribed after the 7th day of stimulation. **Stimulator Starting Dose:** 150 IU. **Trigger:** 10 000 IU hCG (≥2 follicles reached 17 mm). **Luteal support:** 800 mg suppository progesterone, started the day after ovum pick-up by administration of progesterone. **Embryo transfer day:** Day 3.	
**Comparison**	**GnRH Agonist:** Long; Buserelin from D21 of the previous cycle till triggering day. **Pretreatment:** folic acid 1 mg/day before initiation of the induction cycle + low dose OCP on day3 of the previous cycle + doxycycline 100 mg twice daily for the first 10 days of the previous cycle. **Stimulator:** r-FSH, D3 + hMG was prescribed after the 7th day of stimulation. **Stimulator Starting Dose:** 150 IU. **Trigger:** 10 000 IU hCG (≥2 follicles reached 17 mm). **Luteal support:** 800 mg suppository progesterone, started the day after ovum pick-up by administration of progesterone. **Embryo transfer day:** Day 3.	
**Notes**	**Randomization:** Randomization and group assignments were performed sequentially numbered, with no further information. **Allocation:** NI. **Baseline imbalances:** There was a significant difference in the means of age between the two groups; with younger patients in the Antagonist group. **ITT/mITT or PP:** ITT. **Blinded participates:** NI. **Blinded intervention providers:** NI. **Blinded Outcomes assessors:** NI. **Funding:** NI. **OHSS criteria:** reported; Burney et al. 2007 [7]. **Cycle cancellation criteria:** Not reported, they did not mention whether they would have noted any cycle cancellation cases in the study's groups.	
**Study records**	**Hosseini et al., 2010** [8]**.**	

Kurzawa et al., 2008

**Methods**	**Study design:** RCT, single-center, parallel design. **Study duration:** 2004-2006. **Country:** Poland.	
**Participants**	**Inclusion criteria:** PCOS women age ≤ 35 years, BMI < 26 kg/m^2, basal FSH < 12 mIU/ml, negative HBV and HCV virus infection and HIV. PCOS diagnosis was based on Rotterdam criteria (two of the following three manifestations: irregular or absent ovulation, elevated levels of androgenic hormones, and/or enlarged ovaries containing at least 12 follicles each; other conditions with similar signs, such as androgen-secreting tumours or Cushing's syndrome were ruled out).	
	**Exclusion criteria:** Women with ≥ 2 miscarriages, ≥ 3 unsuccessful IVF/ICSI cycles, anatomical abnormalities of the uterus on laparoscopy or hysteroscopy and existence of ovarian cysts.	
	**Baseline characteristics:**	**GnRH Antagonist group: Age:** 31.33±3.91 years. **BMI:** 23.1±1.3 Kg/m^2. **Infertility Duration:** NI. **FSH:** NI. **LH:** NI. **AMH:** NI.
		**GnRH Agonist group: Age:** 30.36±3.40 years. **BMI:** 22.3±1.6 Kg/m^2. **Infertility Duration:** NI. **FSH:** NI. **LH:** NI. **AMH:** NI.

	**Group size**	**GnRH Antagonist group:** 37 women were allocated, 33 women were analyzed.
		**GnRH Agonist group:** 37 women.
	**Lost to follow up/ drop-out**	**GnRH Antagonist group:** None.
		**GnRH Agonist group:** None.
**Intervention**	**GnRH Antagonist:** Flexible; mg Cetrorelix, (≥ 2 follicles reached ≥ 14mm). **Pretreatment:** OCP for a month before starting COS (0.035 mg ethinyl estradiol+0.25 mg norgestimate, Cilest, Janssen-Cilag, Belgium). No oral antidiabetic medications (biguanides or thiazolidinediones). **Stimulator:** r-FSH, D2. **Stimulator Starting Dose:** 150 IU. **Trigger:** 10,000 IU hCG or 250 µg hCG (dominant follicle reached ≥18 mm with the following two ≥16 mm and estradiol level between 1,000 and 4,000 pg/mL). **Luteal support:** 30 mg/day of dydrogesterone and 150 mg/day of progesterone. **Transfer Day:** Day 3.	
**Comparison**	**GnRH Agonist:** Long; Triptorelin from D16–18 of the preceding cycle. **Pretreatment:** OCP for a month before starting COS (0.035 mg ethinyl estradiol+0.25 mg norgestimate, Cilest, Janssen-Cilag, Belgium). No oral antidiabetic medications (biguanides or thiazolidinediones). **Stimulator:** r-FSH, after confirmation of pituitary desensitization (LH <2 mIU/mL and estradiol <40 pg/mL). **Stimulator Starting Dose:** 150 IU. **Trigger:** 10,000 IU hCG or 250 µg hCG (dominant follicle reached ≥18 mm with the following two ≥16 mm and estradiol level between 1,000 and 4,000 pg/mL). **Luteal support:** oral 30 mg/day of dydrogesterone and intravaginal 150 mg/day of progesterone. **Transfer Day:** Day 3.	
**Notes**	**Randomization:** using computer-generated random letters. **Allocation:** Allocations were concealed in opaque sealed envelopes, opened once written informed consent had been obtained. **Baseline imbalances:** NO. **ITT/mITT or PP:** PP. **Blinded participates:** No. **Blinded intervention providers:** PN; In both protocols, only two clinicians and two embryologists, also not blinded to the treatment groups, were involved in the study. **Blinded outcomes assessors:** PN; In both protocols, only two clinicians and two embryologists, also not blinded to the treatment groups, were involved in the study. **Funding:** grant number KBN 2 P05E 034 28 from State Committee for Scientific Research. **OHSS criteria**: Not reported. **Cycle cancellation criteria:** Not reported, all women underwent ET. **Note:** 4 women in the GnRH antagonist group were excluded after randomization, two of them because of insufficient compliance with medication as established by the respective protocol. Further two patients quit the preparations for the treatment without notice.	
**Study records**	**Kurzawa et al., 2008** [9]**, a registry record (ACTRN12607000636459)** [10]**.**	

Lainas et al., 2010

**Methods**	**Study design:** RCT, single-center, parallel design. **Study duration:** Nov 2004-Feb 2008. **Country:** Greece.	
**Participants**	**Inclusion criteria:** PCOS [presence of oligo-ovulation/anovulation and polycystic ovaries], age 18 – 39 years, no endometriotic cyst present, as assessed by transvaginal ultrasound examination, basal FSH ≤ 10 IU/ml.	
	**Exclusion criteria:** Women with known previous poor ovarian response.	
	**Baseline characteristics:** medians (Q25-Q75)	**GnRH Antagonist group: Age:** 31 (28-35) years. **BMI:** 24.6 (20.9-29.3) Kg/m^2. **Infertility Duration:** 3 (2-5) years. **FSH:** 6.2 (4.8-7.5) mIU/ml. **LH:** 5.3 (4.0-7.5) mIU/ml. **AMH:** NI.
		**GnRH Agonist group: Age:** 32 (29-35) years. **BMI:** 23.2 (20.9-25.8) Kg/m^2. **Infertility Duration:** 3 (2-5) years. **FSH:** 6 (4.3-6.9) mIU/ml. **LH:** 5.9 (3.4-7.6) mIU/ml**. AMH:** NI.
	**Group size**	**GnRH Antagonist group:** 110 women.
		**GnRH Agonist group:** 110 women.
	**Lost to follow up/ drop-out**	**GnRH Antagonist group:** None.
		**GnRH Agonist group:** None.
**Intervention**	**GnRH Antagonist:** Flexible; Ganirelix when at least one of the following criteria were fulfilled: (i) the presence of at least one follicle measuring > 14 mm, (ii) serum E2 levels > 600 pg/ml, and (iii) serum LH levels > 10 IU/l (Lainas et al., 2005). **Pretreatment:** OCP from day2 of the preceding menstrual cycle, for 21 days (ethinyl estradiol 0.03 mg+gestodene 0.075 mg, Minulet, Wyeth, Greece). **Stimulator:** r-FSH, D2. **Stimulator Starting Dose:** 150 IU. **Trigger:** 5000 IU hCG administered when 3 follicles reached ≥ 17 mm. **Luteal support:** 600 mg of micronized progesterone was initiated two days after oocyte retrieval. **Transfer Day:** Day 2-3.	
**Comparison**	**GnRH Agonist:** Long; Triptorelin 3 days before discontinuation of the OCP. **Pretreatment: Pretreatment:** OCP from day2 of the preceding menstrual cycle, for 21 days (ethinyl estradiol 0.03 mg+gestodene 0.075 mg, Minulet, Wyeth, Greece). **Stimulator:** r-FSH, when desensitization was achieved (~10–15 days after the initiation of GnRH agonists), as evidenced by plasma E2 levels of ≤ 50 pg/ml, the absence of ovarian follicles and endometrial thickness ≤ 6 mm on transvaginal ultrasound examination. **Stimulator Starting Dose:** 150 IU. **Trigger:** 5000 IU hCG administered (3	

	follicles reached ≥ 17 mm). **Luteal support:** 600 mg of micronized progesterone was initiated two days after oocyte retrieval. **Transfer Day:** Day 2-3.
**Notes**	**Randomization:** by a study nurse at consultation, using a computer-generated randomization list. **Allocation:** NI. **Baseline imbalances:** NO. **ITT/mITT or PP:** ITT. **Blinded Participates:** No. **Blinded intervention providers:** PN; doctors were not blinded to the treatment assigned. **Blinded outcomes assessors:** PN; doctors were not blinded to the treatment assigned. **Funding:** NI. **OHSS criteria:** reported, a modified classification system based on combined criteria previously reported (Golan et al., 1989 [11]; Navot et al., 1992 [12]; Rizk and Aboulghar,1999 [13]). **Cycle cancellation criteria**: reported, Embryo transfer was cancelled and elective embryo cryopreservation was performed in cases of early OHSS, detected 3 days post-oocyte retrieval, that could possibly lead to life-threatening OHSS, or in cases fulfilling one or more of the criteria for hospitalization.
**Study records**	**Lainas et al., 2010** [14]**, Basly et al., 2012** [15]**, a registry record (NCT00417144)** [16]**.**

Mokhtar et al., 2015

**Methods**	**Study design:** RCT, single-center, parallel design. **Study duration:** 2012-2014. **Country:** Iran.	
**Participants**	**Inclusion criteria:** PCOS diagnosis was based on Rotterdam criteria, aged 20-38 years with normal prolactin and thyroid function tests and normal cardiac, hepatic and renal functions who had normal spontaneous onset of puberty and normal sexual development.	
	**Exclusion criteria:** Women with FSH>12 or≥ 2 ART failure or ≥2 first trimester abortion were excluded. Women with ovarian cysts or anatomical abnormality in uterus and cervix or hydrosalpinx.	
	**Baseline characteristics:**	**GnRH Antagonist group: Age:** 28.84±4.44 years. **BMI:** 26.71±3.82 Kg/m2. **Infertility Duration:** 4.45±3.82 years. **FSH:** 6.44±1.62 mIU/ml. **LH:** 6.59±4.23 mIU/ml. **AMH:** NI.
		**GnRH Agonist group: Age:** 27.64±3.65 years. **BMI:** 25.40±4.08 Kg/m2. **Infertility Duration:** 4.87±3.03 years. **FSH:** 6.70±2.22 mIU/ml. **LH:** 7.20±4.29 mIU/ml. **AMH:** NI.
	**Group size**	**GnRH Antagonist group:** 50 women.
		**GnRH Agonist group:** 50 women.
	**Lost to follow up/ drop-out**	**GnRH Antagonist group:** None.
		**GnRH Agonist group:** None.
**Intervention**	**GnRH Antagonist:** Flexible; Cetrorelix (≥ 2 follicles reached 13-14). **Pretreatment:** OCP for 21 days from the preceding menstrual cycle (ethinyl estradiol 0.03 mg + levonorgestrel 0.150 mg; Ovocept LD, Abureihan, Iran). **Stimulator:** r-FSH, D3. **Stimulator Starting Dose:** 150 to 225 IU. **Trigger:** 10000 IU hCG; when ≥ 2 follicles with a diameter ≥ 17 mm were observed. **Luteal support:** progesterone (Progesterone in Oil 50 mg/mL) from the day after ovum pick-up and 100 mg after embryo transfer. **Transfer Day:** Day 3.	
**Comparison**	**GnRH Agonist:** Long; Buserelin from D21 of the preceding cycle. **Pretreatment: Pretreatment:** OCP for 21 days from the preceding menstrual cycle (ethinyl estradiol 0.03 mg + levonorgestrel 0.150 mg; Ovocept LD, Abureihan, Iran). **Stimulator:** r-FSH; when pituitary desensitization was achieved (absence follicle diameter >10 mm and estradiol level <40 pg/ml). **Stimulator Starting Dose:** 150 to 225 IU. **Trigger:** 10000 IU hCG; when ≥ 2 follicles with a diameter ≥ 17 mm were observed. **Luteal support:** progesterone (Progesterone in Oil 50 mg/mL) from the day after ovum pick-up and 100 mg after embryo transfer. **Transfer Day:** Day 3.	
**Notes**	**Randomization:** using computer-generated random letters. **Allocation:** NI. **Baseline imbalances:** No. **ITT/mITT or PP:** ITT/mITT. **Blinded participates:** Yes; based on the information provided by the author "patients completed consent form and did not know which group they were in". **Blinded intervention providers:** PN, (Registry record). **Blinded outcomes assessors:** PN, (Registry record). **Funding:** Avicenna Infertility Treatment Center. **OHSS criteria:** reported, Golan 2009 [17]. **Cycle cancellation criteria:** Not reported.	
**Study records**	**Mokhtar et al., 2015** [18]**, a registry record (IRCT2012120311653N1)** [19]**.**	

Shin et al., 2018

**Methods**	**Study design:** RCT, multi-center, parallel design. **Study duration:** Feb 2011-Dec 2016. **Country:** Korea.
**Participants**	**Inclusion criteria:** PCOS aged 20–40 years; presence of both ovaries without ovarian tumours; a normal uterine cavity as assessed through an ultrasound scan, hysterosalpingograms, or hysteroscopy; normal renal, liver and haematological indices; normal thyroid function; and normal prolactin levels. PCOS diagnosis was based on Rotterdam criteria, so all women had at least two of the three following characteristics: (1) oligo- or anovulation, (2) clinical and/or biochemical signs of hyperandrogenism, and (3) polycystic ovaries with the exclusion of other possible etiologies.

	**Exclusion criteria:** Women with extrauterine pregnancy or abortion in the past 3 months; abnormal gynaecological bleeding of undetermined origin; a congenital uterine anomaly; congenital adrenal hyperplasia; androgen-secreting tumours; Cushing syndrome; any contraindication for recombinant follicle-stimulating hormone (r-FSH), GnRH analogue, or human chorionic gonadotropin (hCG) administration; and severe male factor infertility.	
	**Baseline characteristics:**	**GnRH Antagonist group: Age:** 32.7±3.2 years. **BMI:** 23.6±5.3 Kg/m^2. **Infertility Duration:** NI. **FSH:** 6.3±2.4 mIU/ml. **LH:** 7.5±2.8 mIU/ml. **AMH:** 9.8±5.6 ng/ml. (information was provided by the author)
		**GnRH Agonist group: Age:** 34.2±3.4 years. **BMI:** 22.9±2.9 Kg/m^2. **Infertility Duration:** NI. **FSH:** 6.5±1.5 mIU/ml. **LH:**7.5±4.9 mIU/ml. **AMH:** 8.5±3.9 ng/ml. (information was provided by the author)
	**Group size**	**GnRH Antagonist group:** 14 women were randomized (information provided by the author), but 11 women were analyzed.
		**GnRH Agonist group:** 13 women were randomized (information provided by the author), but 11 women were analyzed.
	**Lost to follow up/ drop-out**	**GnRH Antagonist group:** 3 women discontinued participation after the initial agreement (information provided by the author).
		**GnRH Agonist group:** 2 women discontinued participation after the initial agreement (information provided by the author).
**Intervention**	**GnRH Antagonist:** Fixed; Cetrorelix from S6 (D6 of stimulation) through the triggering day. **Pretreatment:** OCP from Day5 of the preceding menstrual cycle, for 21 days (ethinyl estradiol 0.03 mg+cyproterone acetate 2 mg, Diane 35, Bayer AG, Leverkusen, Germany; other OCP were also permitted). **Stimulator:** r-FSH, D3. **Stimulator Starting Dose:** 150 (150–225). **Trigger:** 250 μg recombinant hCG or 10,000 IU urinary hCG was administered (≥ 2 follicles reached 17 mm in diameter). **Luteal support:** vaginal progesterone was started on the day of oocyte retrieval and was continued for up to 8 weeks. **Transfer Day:** 2-5 Day.	
**Comparison**	**GnRH Agonist:** Long; Triptorelin from D18. **Pretreatment:** OCP from Day5 of the preceding menstrual cycle, for 21 days (ethinyl estradiol 0.03 mg+cyproterone acetate 2 mg, Diane 35, Bayer AG, Leverkusen, Germany; other OCP were also permitted). **Stimulator:** r-FSH, D3. **Stimulator Starting Dose:** 225 (150–300). **Trigger:** 250 μg recombinant hCG or 10,000 IU urinary hCG was administered (≥ 2 follicles reached 17 mm in diameter). **Luteal support:** vaginal progesterone was started on the day of oocyte retrieval and was continued for up to 8 weeks. **Transfer Day:** 2-5 Day.	
**Notes**	**Randomization and allocation:** using a web-based system provided by the Medical Research Collaborating Center of Seoul National University Hospital. Stratified randomization was used (stratification according to the institution, block randomization with a randomly selected block size). The investigator was blind to the randomization process, including the size of each block. **Baseline imbalances:** PN; AMH was slightly lower in the agonist group but the differences were not significant. **ITT/mITT or PP:** mITT. **Blinded participates:** NO. **Blinded intervention providers:** No; “doctors were not blinded to the treatment assigned” and the information was confirmed by the author. **Blinded outcomes assessors:** No; “doctors were not blinded to the treatment assigned” and the information was confirmed by the author. **Funding:** This study was supported by a grant from Merck Ltd., Republic of Korea. The funder had no role in the design, collection, analysis or interpretation of this study. No other potential conflict of interest relevant to this article was reported. **OHSS criteria:** reported, Golan 2009 [17]. **Cycle cancellation criteria:** based on the information provided by the author; “since it was a multi-center RCT, they could not have a unified criterion for every possible circumstances. Hence the criteria for cycle cancellation were up to clinicians to decide".<br>The following information was provided by the author:<br>Gonadotropin consumption (mean ± SD) IUs: [in GnRH antagonist group (n=11): 1544.3 ± 488.0, in GnRH agonist group (n=11): 1963.6 ± 565.1]. Stimulation duration (mean ± SD) days: [in GnRH antagonist group (n=11): 9.2 ± 1.5, in GnRH agonist group (n=11): 9.9 ± 2.7]. E2 levels on hCG day (mean ± SD) pg/ml: [in GnRH antagonist group (n=11): 3169.1 ± 3386.9, in GnRH agonist group (n=10): 2242.4 ± 1254.1, data of one woman of the GnRH agonist group were missing due to documentation reasons]. Endometrial thickness on hCG day (mean ± SD) mm: [in GnRH antagonist group (n=8): 8.1 ± 4.7, in GnRH agonist group (n=9): 6.7 ± 5.4, data of three women of the GnRH antagonist group and two women of the GnRH agonist group were missing due to documentation reasons]. No. of retrieved oocytes (mean ± SD): [in GnRH antagonist group (n=11): 18.3 ± 17.4, in GnRH agonist group (n=11): 19.1 ± 13.0].	
**Study records**	**Shin et al., 2018** [20]**, a registry record (NCT01402336)** [21]**.**	

Trenkic et al., 2016

**Methods**	**Study design:** RCT, single-center, parallel design. **Study duration:** 2013-2014. **Country:** Serbia.	
**Participants**	**Inclusion criteria:** PCOS diagnosed based on Rotterdam criteria, aged 18-39 years, BMI 18-30 Kg/m^2. Each patient could participate in the study only once.	
	**Exclusion criteria:** abnormalities of the uterine cavity, dysfunction of thyroid or abnormal prolactin levels, ovarian cysts as well as severe disturbances of spermatogenesis of the partner, requiring the ICSI method.	
	**Baseline characteristics:**	**GnRH Antagonist group: Age:** 31.36±4.02 years. **BMI:** 23.22±3.16 Kg/m^2. **Infertility Duration:** NI. **FSH:** 5.53±1.85 mIU/ml. **LH:** 6.84±1.96 mIU/ml. **AMH:** 6.73±2.88 ng/ml.
		**GnRH Agonist group: Age:** 31.20±3.98 years. **BMI:** 23.16±3.03 Kg/m^2. **Infertility Duration:** NI. **FSH:** 5.40±1.74 mIU/ml. **LH:** 7.44±3.28 mIU/ml. **AMH:** 7.13±3.57 ng/ml.
	**Group size**	**GnRH Antagonist group:** 45 women.
		**GnRH Agonist group:** 45 women.
	**Lost to follow up/ drop-out**	**GnRH Antagonist group:** None.
		**GnRH Agonist group:** None.
**Intervention**	**GnRH Antagonist:** Flexible; Cetrorelix (the lead follicle reached the size 14 mm and/or E2 levels > 300 pg/mL) until and including the day of triggering. **Pretreatment:** OCP from Day2 of the preceding cycle, for 21 days. **Stimulator:** r-FSH, D2. **Stimulator Starting Dose:** 150 IU. **Trigger:** 10,000 IU hCG (3 follicles reached the size ≥17 mm or the dominant follicle ≥18 mm with the following two ≥16 mm). **Luteal support:** 600 mg micronized Progesterone per day. **Transfer day:** Day 3.	
**Comparison**	**GnRH Agonist:** Long; Triptorelin on D21 of the menstrual cycle. **Pretreatment:** OCP from Day2 of the preceding cycle, for 21 days. **Stimulator:** r-FSH, after confirmation of pituitary desensitization (LH <2 mIU/mL and estradiol <20 pg/mL). **Stimulator Starting Dose:** 150 IU. **Trigger:** 10,000 IU hCG (3 follicles reached the size ≥17 mm or the dominant follicle ≥18 mm with the following two ≥16 mm). **Luteal support:** 600 mg micronized Progesterone per day. **Transfer day:** Day 3.	
**Notes**	**Randomization:** computer-generated randomization. **Allocation:** NI. **Baseline imbalances:** NO. **ITT/mITT or PP**: ITT/mITT. **Blinded participates:** No. **Blinded intervention providers:** PN; doctors were not blinded to the treatment assigned. **Blinded outcomes assessors:** PN; doctors were not blinded to the treatment assigned. **Funding:** The authors certify that have NO affiliations with or involvement in any organization or entity with any financial interest, and have non-financial interest in the subject matter or materials discussed in the manuscript. **OHSS criteria:** reported, Golan 1989 [11]. **Cycle cancellation criteria:** Not reported.	
**Study records**	**Trenkic et al. 2016** [22]**.**	

AMH: anti- müllerian hormone, **BMI**: Body Mass Index, **E2**: Estradiol, **ET**: Embryo Transfer, **FSH**: follicle-stimulation hormone**, GnRH**: Gonadotropin-releasing hormone, **HBV**: Hepatitis B Virus, **hCG**: human chorionic gonadotropin**, HCV**: Hepatitis C Virus**, HIV**: Human-immunodeficiency Virus, **hMG**: human menopausal gonadotropin, **ICSI**: Intra-Cytoplasmic Sperm Injection, **IM**: Intra-muscular. **ITT**: Intention-to-treat analysis**, IVF**: In-vitro Fertilization, **LH**: luteinizing hormone, **mITT**: modified Intention-to-treat analysis**, NI**: No Information, **OCP**: oral contraceptive pills, **OHSS**: Ovarian hyper-stimulation syndrome**, PCOS**: Polycystic ovary syndrome, **PN**: Probably No, **PP**: Per Protocol analysis, **PY**: Probably Yes, **RCT**: Randomized controlled trial, **r-FSH**: recombinant follicle-stimulating hormone. $\mathbf{S_{(number)}}$: Stimulation day number.

References:

1. Page, M. J. *et al.* The PRISMA 2020 statement: an updated guideline for reporting systematic reviews. *BMJ* **372**, (2021).
2. Bahçeci, M. *et al.* Use of a GnRH antagonist in controlled ovarian hyperstimulation for assisted conception in women with polycystic ovary disease: A randomized, prospective, pilot study. *J. Reprod. Med. Obstet. Gynecol.* **50**, 84–90 (2005).
3. Choi, J. W. *et al.* Efficacy of controlled ovarian hyperstimulation using GnRH antagonist in women with polycystic ovary syndrome undergoing IVF-ET. *Korean J Obs. Gynecol* **48**, 716–725 (2005).
4. Ghaebi, N. K. *et al.* Pregnancy outcomes in PCOS patients undergoing IVF with long GnRH agonist protocol versus flexible GnRH antagonist. *Iran. J. Obstet. Gynecol. Infertil.* **21**, 1–9 (2018).
5. Haydardedeoglu, B., Kilicdag, E. B., Parlakgumus, A. H. & Zeyneloglu, H. B. IVF/ICSI outcomes of the OCP plus GnRH agonist protocol versus the OCP plus GnRH antagonist fixed protocol in women with PCOS: a randomized trial. *Arch. Gynecol. Obstet.* **286**, 763–769 (2012).
6. ClinicalTrials.gov. Bethesda (MD): National Library of Medicine (US). NCT01354275. In Vitro Fertilization outcomes of two treatment protocols in women with Polycystic Ovary Syndrome (PCOS and IVF). (2011).
7. Burney, R., Schust, D. & Yao, M. Infertility. in *Berek and Novak's Gynecology* (ed. Berek JS) 1185–1276 (Lippincott Williams & Wilkins, 2007).
8. Hosseini, M. A. *et al.* Comparison of gonadotropin-releasing hormone agonists and antagonists in assisted reproduction cycles of polycystic ovarian syndrome patients. *J. Obstet. Gynaecol. Res.* **36**, 605–610 (2010).
9. Kurzawa, R., Ciepiela, P., Baczkowski, T., Safranow, K. & Brelik, P. Comparison of embryological and clinical outcome in GnRH antagonist vs. GnRH agonist protocols for in vitro fertilization in PCOS non-obese patients. A prospective randomized study. *J. Assist. Reprod. Genet.* **25**, 365–374 (2008).
10. Australian New Zealand Clinical Trials Registry: Sydney (NSW): NHMRC Clinical Trials Centre, U. of S. (Australia). ACTRN12607000636459. A prospective randomized study comparing clinical and embryological outcomes in controlled ovarian hyperstimulation protocols with GnRH antagonist and GnRH agonist for in vitro fertilization in PCOS non-obese patients. (2007).
11. Golan, A. *et al.* Ovarian hyperstimulation syndrome: an update review. *Obstet. Gynecol. Surv.* **44**, 430–440 (1989).
12. Navot, D., Bergh, P. A. & Laufer, N. Ovarian hyperstimulation syndrome in novel reproductive technologies: prevention and treatment. *Fertil. Steril.* **58**, 249–261 (1992).
13. Rizk, B. & Aboulghar, M. Classification, pathophysiology and management of ovarian hyperstimulation syndrome. in *In-Vitro Fertilization and Assisted Reproduction* (ed. Brinsden, P.) 131–155 (The Parthenon Publishing Group, 1999).
14. Lainas, T. G. *et al.* Flexible GnRH antagonist protocol versus GnRH agonist long protocol in patients with polycystic ovary syndrome treated for IVF: A prospective randomised controlled trial (RCT). *Hum. Reprod.* **25**, 683–689 (2010).
15. Basly, M. *et al.* Flexible Gnrh antagonist protocol versus Gnrh agonist long protocol in patients with polycystic ovary syndrome treated for IVF: A prospective randomised controlled trial (RCT). *Internet J. Gynecol. Obstet.* **16**, (2012).
16. ClinicalTrials.gov. Bethesda (MD): National Library of Medicine (US). NCT00417144. Comparison between GnRH agonist and GnRH antagonist protocols of ovarian stimulation in PCOS patients. (2006).
17. Golan, A. & Weissman, A. Update on prediction and management of OHSS. A modern classification of OHSS. *Reprod. Biomed. Online* **19**, 28–32 (2009).
18. Mokhtar, S. *et al.* ART outcomes in GnRH antagonist protocol (flexible) and long GnRH agonist protocol during early follicular phase in patients with polycystic ovary syndrome: A randomized clinical trial. *J. Reprod. Infertil.* **16**, 148–154 (2015).
19. Iranian Registry of Clinical Trials. IRCT2012120311653N1. Comparing IVF outcomes in GnRH antagonist protocol during early and late follicular phase and GnRH antagonist protocol (flexible) and long GnRH agonist protocol in patients with polycystic ovary syndrome. (2013).
20. Shin, J. J. *et al.* Early gonadotropin-releasing hormone antagonist protocol in women with polycystic ovary syndrome: A preliminary randomized trial. *Clin. Exp. Reprod. Med.* **45**, 135–142 (2018).

21. ClinicalTrials.gov. Bethesda (MD): National Library of Medicine (US). NCT01402336. GnRH antagonist versus GnRH agonist in Polycystic Ovary Syndrome during In Vitro Fertilization - Embryo Transfer. (2011).
22. Trenkić, M. S. *et al.* Flexible GnRH antagonist protocol vs. long GnRH agonist protocol in patients with polycystic ovary syndrome treated for IVF: Comparison of clinical outcome and embryo quality. *Ginekol. Pol.* **87**, 265–270 (2016).
